# Investigations on genomic, topological and structural properties of diguanylate cyclases involved in *Vibrio cholerae* biofilm signalling using *in silico* techniques: Promising drug targets in combating cholera

**DOI:** 10.1016/j.crstbi.2025.100166

**Published:** 2025-04-09

**Authors:** Tuhin Manna, Subhamoy Dey, Monalisha Karmakar, Amiya Kumar Panda, Chandradipa Ghosh

**Affiliations:** a Deparment of Human Physiology, Vidyasagar University, Midnapore, West Bengal, India; b Department of Chemistry, Vidyasagar University, Midnapore, West Bengal, India; c Centre for Life Sciences, Vidyasagar University, Midnapore, West Bengal, India; d Rani Rashmoni Green University, Singur, West Bengal, India

**Keywords:** *Vibrio cholerae*, Structural bioinformatics, Diguanylate cyclases, Biofilm, Cyclic-di-GMP

## Abstract

During various stages of its life cycle, *Vibrio cholerae* initiate biofilm signalling cascade. Intercellular high level of the signalling nucleotide 3′-5′ cyclic dimeric guanosine monophosphate (c-di-GMP), synthesized by diguanylate cyclases (DGCs) from its precursor molecule GTP, is crucial for biofilm formation. Present study endeavours to *in silico* approaches in evaluating genomic, physicochemical, topological and functional properties of six c-di-GMP regulatory DGCs (CdgA, CdgH, CdgK, CdgL, CdgM, VpvC) of *V. cholerae*. Genomic investigations unveiled that codon preferences were inclined towards AU ending over GC ending codons and overall GC content ranged from 44.6 to 49.5 with codon adaptation index ranging from 0.707 to 0.783. Topological analyses deciphered the presence of transmembrane domains in all proteins. All the DGCs were acidic, hydrophilic and thermostable. Only CdgA, CdgH and VpvC were predicted to be stable during *in vitro* conditions. Non-polar amino acids with leucine being the most abundant amino acid among these DGCs with α-helix as the predominant secondary structure, responsible for forming the transmembrane regions by secondary structure analysis. Tertiary structures of the proteins were obtained by computation using AlphaFold and trRosetta. Predicted structures by both the servers were compared in various aspects using PROCHECK, ERRAT and Modfold8 servers. Selected 3D structures were refined using GalaxyRefine. InterPro Scan revealed presence of a conserved GGDEF domain in all DGCs and predicted the active site residues in the GGDEF domain. Molecular docking studies using CB-DOCK 2 tool revealed that among the DGCs, VpvC exhibited highest affinity for GTP (−5.6 kcal/mol), which was closely followed by CdgL (−5.5 kcal/mol). MD simulations depicted all DGC-GTP complexes to be stable due to its considerably low eigenvalues. Such studies are considered to provide maiden insights into the genomic and structural properties of *V. cholerae* DGCs, actively involved in biofilm signalling systems, and it is projected to be beneficial in the discovery of novel DGC inhibitors that can target and downregulate the c-di-GMP regulatory system to develop anti-biofilm strategies against the cholera pathogen.

## Highlights

• Genomic & structural analyses of biofilm regulatory *V. cholerae* DGCs were conducted.
• AU ending codons were preferred over GC ending codons in most DGCs.
• DGCs were acidic, hydrophilic proteins with transmembrane regions.
• Comparative analysis of DGC structures with AlphaFold and trRosetta.
• CdgL and VpvC had high affinity towards GTP.

## Graphical abstract

**Figure:**
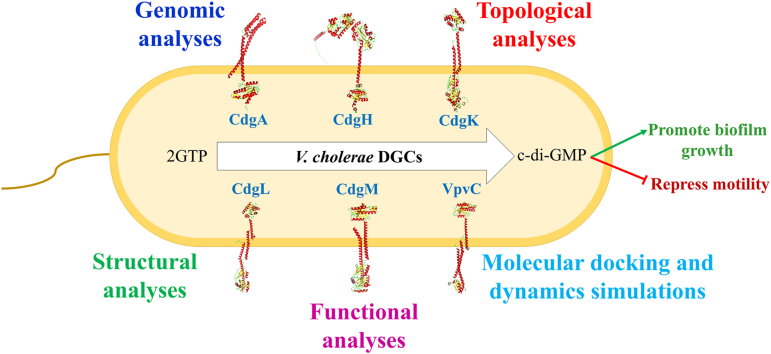

## 1 Introduction

Cholera is a significant public health concern known for its ability to cause large-scale outbreaks and it is considered as an emerging and re-emerging infectious disease (Mavian et al., 2023). The causative agent *i.e. Vibrio cholerae* can withstand a number of environmental challenges by generating variety of adaptive responses, which often entail unique patterns of gene expression to produce biofilm. Biofilms are microbial communities, formed by the microorganisms themselves, consist of an extracellular matrix. According to the National Institutes of Health (NIH), microbial biofilms are associated with 60–80 % of all microbial illnesses (Jamal et al., 2018). In case of *V. cholerae*, both biofilms and cells that can disperse from a preformed biofilm are more pathogenic than planktonic, free-living cells (Tamayo et al., 2010). *V. cholerae*, residing in aquatic reservoirs, initiates the biofilm formation on chitinous surfaces (Walker et al., 2023) using a variety of signal transduction mechanisms to sense surrounding environmental conditions (Biswas et al., 2019). Signalling nucleotide, crucial for this pathway, is 3′-5′ cyclic dimeric guanosine monophosphate (c-di-GMP). C-di-GMP, first identified as an allosteric regulator of cellulose synthesis in *Gluconacetobacter xylinus* (Ross et al., 1987) functions as a global second messenger in signal transduction pathways to control various cellular functions in bacteria including biofilm formation, motility, and cell differentiation in majority of bacteria including *V. cholerae* (Conner et al., 2017; Kim and Harshey, 2016). So far, it has been found that c-di-GMP can bind to various receptor proteins, or riboswitches, and regulates biofilm formation, motility, virulence, *etc*. (Schulze et al., 2021). Intercellular high level of c-di-GMP enhances biofilm formation by triggering the expressions of Vibrio polysaccharide (VPS) biosynthesis genes (Tischler and Camilli, 2005). *V. cholerae* are known to possesses 62 genes that encode proteins with domains predicted to be involved in governing intracellular c-di- GMP levels (Galperin, 2004). Diguanylate cyclases (DGCs) are a category of enzymes having GGDEF domains that synthesize c-di-GMP from GTP (Hengge, 2009; Conner et al., 2017). In *V. cholerae* DGCs are integral to the formation and regulation of biofilms, which are critical for bacterial survival and pathogenicity. DGCs regulate the transition between planktonic and biofilm lifestyles, enhancing bacterial infectivity and adaptability (Biswas et al., 2020). High activation of DGCs resulting in higher c-di-GMP levels correlate with increased biofilm formation, which is associated with enhanced persistence. This is particularly important for the survival of *V. cholerae* in aquatic environments as well as during host infections (Silva and Benitez, 2016; Conner et al., 2017).

Analyses of biofilm formation and *vps* gene expression of *V. cholerae* demonstrated the key DGCs that regulate biofilm formation are CdgA (VCA0074), CdgH (VC1067), CdgK (VC1104), CdgL (VC2285), CdgM (VC1376), and VpvC (VC2454) in various environmental conditions (Beyhan et al., 2008; Conner et al., 2017). CdgA, along with CdgL, promote c-di-GMP synthesis in the absence of flagellar protein FlaA and are involved in flagellum-dependent biofilm regulatory (FDBR) response and CdgA also has the largest hierarchical effect on VPS expression (Wu et al., 2020). CdgL contributes to the basal c-di-GMP pool, loss of CdgL gene results in reduced cytoplasmic c-di-GMP levels and thereby exhibits less biofilm related gene expression*.* CdgH is required for optimal VPS production and strains lacking CdgH have a reduced capacity to form biofilm (Shikuma et al., 2012). It also plays major roles in c-di-GMP synthesis by responding to the presence of bile acids (Koestler and Waters, 2014). CdgH and CdgM, possess sensory domains that may be involved in sensing bile acids. Interestingly, CdgH and CdgM are also involved in the response to temperature, indicating that these proteins are part of multiple c-di-GMP signalling pathways (Townsley and Yildiz, 2015). CdgK is actively involved in upregulation of c-di-GMP. It is homologous to *casA* DGC of *V. fischeri*, which inhibits motility and drives cellulose-dependent biofilm formation. CdgK is predicted to have similar type function in *V. cholerae* (Townsley and Yildiz, 2015). Earlier reports have also shown that another DGC, VpvC is required for increased biofilm formation capacity in the rugose variant of wild type *V. cholerae* (Beyhan and Yildiz, 2007). In frame deletion of *vpvC* resulted into conversion of rugose colonies to smooth colonies with downregulation of *vps* transcription followed by low biofilm formation (Beyhan et al., 2008). Hence, these DGCs are involved in maintaining cellular c-di-GMP levels where they additively contribute to biofilm formation and *vps* gene expressions (Shikuma et al., 2012).

DGCs are associated with different levels biofilm related signalling pathways, but their codon usage preference and structural properties are not very well understood. The degeneracy of the genetic code allows same amino acid to be expressed by multiple codons (except Met and Trp) (Chaudhary et al., 2023), regarded as synonymous codons. But usage of synonymous codons are not random, leading to codon usage bias (CUB). Codon biasness often affected by certain factors *viz.,* environmental conditions, genetic drift, base mutations, tRNA abundance, and gene expression levels which is critical for various cellular processes like transcription, mRNA stability, translation efficiency and accuracy, and protein expression and co-translational folding (Ling et al., 2024). Thus, studies regarding codon usage bias is considered useful to understand expression probability, evolutionary aspects of the DGCs. Codon usage patterns play significant roles in drug design by influencing protein expression, structure, and function, which are critical for developing effective therapeutic agents. Synonymous codon usage bias also affects the translation efficiency and protein folding, thereby impacting the drug efficacy (Athey et al., 2017). Regarding structural characteristics, majority of the protein structures in the Protein Data Bank have been characterised using X-ray crystallography (Hunter et al., 2011). As these DGCs might belong to the category of transmembrane (TM) proteins, traditional X-ray crystallography studies are very difficult to execute (Lacapère et al., 2007). Membrane proteins, isolated in detergent-bound form, pose difficulties for crystallization due to the necessity for extensive intermolecular contacts. The detergents used in purification typically exhibit repulsions, hindering effective protein contacts (Otzen, 2002). The limitation of the NMR spectroscopy is that only smaller protein structures (12 KDa) can be determined. Larger proteins with molecular weight higher than 25 kDa are difficult to determine solely by NMR spectroscopy (Wüthrich, 2001). Among these DGCs, only the periplasmic portion of the cdgH has been studied (Xu et al., 2017). To address this problem, bioinformatic techniques can provide a speedy, cost-effective, and dependable solution. In the advancing field of computational biology, *in silico* proteomic characterisation plays an essential role for data acquisition and processing to unravel protein structure-function relationships.

WHO has set a target to reduce cholera mortality up to 90 % by 2030 as a part of broader efforts to combat emerging and re-emerging infectious diseases (Bhattacharya et al., 2022). As biofilm production is a key mechanism for survival of *V. cholerae* in environment as well as human intestines different scientific studies were conducted in order to inhibit the biofilm formation or destroy preformed biofilms. One of the most promising anti-biofilm approach is developing strategies to inhibit DGCs to supress c-di-GMP mediated biofilm formation in diverse bacterial species (Caly et al., 2015; Fernicola et al., 2016; Cho et al., 2020). Moreover, studies also report c-di-GMP produced due to DGC activations increases biofilm productions to increase phenotypical antibiotic resistance and tolerance hence DGCs could be considered as an attractive drug target for controlling biofilm centred chronic infections (Christen et al., 2019). Thus, DGCs have been identified as a potential drug target to combat biofilm mediated bacterial infections due to its conserved domain structure and exclusive presence in bacterial systems (Ghosh et al., 2023) and *V. cholerae* is considered as one of the prominent model organisms to study biofilm and its signalling pathway (van Kessel and Camilli, 2024). Prior studies on *V. cholerae* including reports from present research group revealed mutations/absence of these studied six DGCs results in formation of poor quality of biofilm matrix production in *V. cholerae* (Conner et al., 2017; Manna et al., 2024). Besides, the present research group is actively engaged in studying different aspects of *V. cholerae*, including molecular epidemiology, biofilm signalling, developing antibiofilm strategies, etc. (Dua et al., 2018; Manna et al., 2024; Biswas et al., 2019; Guchhait et al., 2022). Prior to the experimental works, it is believed that the theoretical approaches can efficiently provide the information on the underlying mechanism that would eventually be helpful in framing the experimental set up more efficiently. In this context, the present study aims to evaluate and compare the genomic, physicochemical, topological, structural properties of six DGCs, *viz.*, CdgA, CdgH, CdgK, CdgL, CdgM and VpvC followed by molecular docking and simulations. It is expected, the genomic and structural elucidations of these biofilm influencing DGCs will be useful to design potential inhibitors in targeting *V. cholerae* biofilm signalling pathway.

## 2 Methodology

### 2.1 Retrieval of DGC sequences

*Vibrio cholerae* N16961 strain was selected as a model organism for the present study as it belonged to the category of O1-El tor serogroup, responsible for the ongoing seventh cholera pandemic and it also shares very high sequence similarities with endemic causing *V. cholerae* serogroup 0139 responsible for cholera endemics in southeast Asia. Sequences of six important biofilm regulatory DGCs *viz*., CdgA, CdgH, CdgK, CdgL, CdgM, and VpvC were idendified with their previously described locus tags VCA0074, VC1067, VC1104, VC2285, VC1376, VC2454 respectively. The gene sequences and translated protein sequences were downloaded in fasta format. Only the CdgA (VCA0074) sequences was retrieved from chromosome II sequence whereas the rest were fetched from the chromosome I sequence of *Vibrio cholerae* N16961 deposited in NCBI (https://www.ncbi.nlm.nih.gov/). For docking studies, the 3D structure of GTP molecule (DrugBank ID: DB04137) was downloaded from protein data bank (https://www.rcsb.org/) in sdf format.

### 2.2 Codon compositional analysis

CAIcal server (https://genomes.urv.es/CAIcal/) server was used to calculate various genomic parameters like GC content and relative synonymous codon usage (RCSU) (Puigbò et al., 2008). A3 %, T3 %, G3 %, C3 %, GCs (GC1 %, GC2 %, GC3 %, GC %), and Nc values of studied six DGCs of *V. cholerae* were evaluated. The RSCU value for each codon was derived by the following equation.

(1) $${RSCU}_{ij}=\frac{X_{ij}}{\frac{1}{ni}\sum_{j=1}^{ni}X_{ij}}$$

where, X_ij_ is the frequency of the jth codon for the ith amino acid, and n_i_ is the number of codons for the ith amino acid (ith codon family). For analyses of statistical significance of synonymous codons data one tail *t*-test for two independent RSCU values was carried out depending on G and C, A and U ending codons of those amino acids containing a minimum of four degenerative codons that only altered at the third position. At *P* < 0.05 the *t*-test values ≥+1.960 and ≤1.960 were considered to be significant (Dey et al., 2022).

### 2.3 ENc plot analysis

The effective number of codons (ENc) was employed to assess the overall distributions of GC content at the first, second, and third positions of codons for the corresponding gene sequences, thereby ensuring a comprehensive examination of synonymous codon utilization. The ENc metric is generally characterized by a pronounced bias in codon usage and vice versa. ENc and GC3s plot was derived to determine the influence of a predominant mutation on the patterns of codon usage. The expected ENc values for each GC3 were determined under H_0_ (Null hypothesis, *i.e*. no selection) utilising the equation displayed below (Dey et al., 2022; Prabha et al., 2017).

(2) $$ENc=2+S+\left(\frac{29}{S^{2}+{\left(1-S\right)}^{2}}\right)$$

where, 's' denotes GC content at the third codon location.

### 2.4 Neutrality plot and parity rule 2 (PR2) bias plot

Effects of mutational pressure and natural selection on codon usage is studied by neutrality plot analysis by plotting the GC3s versus average of GC1s and GC2s values in X and Y axis respectively (Chaudhary et al., 2023). Neutrality plotting is widely used to analyse the effects of natural selection and mutation pressure on codon usage (Yu et al., 2021). To analyse role of natural selection versus mutational pressure the correlation analysis using Pearson's correlation (at a significance level of *P* < 0.05) was performed between GC12s and GC3s values of the DGCs.

The Parity Rule 2 (PR2) bias plot is employed to evaluate the influences of natural selection and mutation pressure on the third codon position. Nucleotide composition of the four bases *i.e.* adenine (A), Uracil (U), cytosine (C), and guanine (G)at the third codon position of each gene was performed to derive the GC bias [G3 %/(G3 + C3) %] and the AU bias [A3 %/(A3 + T3) %]. A graphical representation was constructed by plotting GC bias against AU bias in the X and Y coordinates respectively to depict the association between purines (A and G) and pyrimidines (C and T) within genes. The central point in the plot signifies the state of equilibrium (A = T, C = G), wherein both coordinates have the value of 0.5 (Ling et al., 2024).

### 2.5 Codon adaptation index

Codon adaptation index (CAI) was used to measures the adaptability of DGC codons of *V. cholerae* strains by using dataset downloaded from Codon Usage Database (https://www.kazusa.or.jp/codon/) (Nakamura et al., 2000) and used as reference to calculate the CAI using CAIcal service (https://genomes.urv.es/CAIcal/). This metric assesses the bias present in synonymous codon usage within a specific arrangement of DNA sequences by juxtaposing the synonymous codon frequency from a reference set against the synonymous codon frequency of a query sequence. This parameter is useful to assess the extent to which selective pressures have influenced codon usage patterns (Nakamura et al., 2000) The value of CAI generally ranges between 0 and 1, with 1 signifying better adaptation likelihood and gene compatibility (Dey et al., 2022).

### 2.6 Subcellular localization and topological analysis

Cellular localization of the target c-di-GMP regulatory DGCs were predicted by using web servers like: PSORTdb v.4.0 (https://db.psort.org/) (Lau et al., 2021), Gneg-PLoc (http://www.csbio.sjtu.edu.cn/bioinf/Gneg-multi/) (Shen and Chou, 2010), LocTree3 (https://rostlab.org/services/loctree3/) (Goldberg et al., 2014).

To find out the transmembrane regions, topological analysis of each protein was carried out using various online computational tools, *viz.*, Deep TMHMM (https://dtu.biolib.com/DeepTMHMM) (Hallgren et al., 2022), MEMSAT 2 (http://www.sacs.ucsf.edu/cgi-bin/memsat.py) (Jones et al., 1994), TOPCONS (https://topcons.net/) (Tsirigos et al., 2015), CCTOP (https://cctop.ttk.hu/), (Dobson et al., 2015), Phobius (https://www.ebi.ac.uk/Tools/pfa/phobius/), (Käll et al., 2007) TMSEG (https://rostlab.org/owiki/index.php/Tmseg), (Bernhofer et al., 2016). For the visualization of transmembrane regions in 3D structures MembraneFold server was used (Gutierrez et al., 2022). Membrane fold helps to visualise the transmembrane regions predicted by DeepTMHMM. For detection of presence of signal peptide Deep TMHMM and SignalP v6.0 (https://services.healthtech.dtu.dk/services/SignalP-6.0/) (Teufel et al., 2022) were used.

### 2.7 Crystallization analyses

Crystallization score of each DGC was predicted by XtalPred server (https://xtalpred.godziklab.org/XtalPred-cgi/xtal.pl). It is useful to assess protein crystallizability (Slabinski et al., 2007). Crystallization possibility of the submitted protein sequence was assessed by comparing its predicted biochemical and biophysical properties. Amino acid sequences of all six target DGCs were submitted to XtalPred server to analyse their crystallization scores.

### 2.8 Physicochemical properties

Physicochemical characterization of the retrieved sequences of c-di-GMP regulatory proteins were performed using *in silico* ExPASy-ProtParam tool (http://web.expasy.org/protparam/) (Gasteiger et al., 2005). Different parameters, *viz.*, number of amino acids theoretical isoelectric point (pI), molecular weight (MW), and instability index (II), aliphatic index (AI), grand average of hydropathicity (GRAVY) values were computed.

### 2.9 Secondary structure evaluation

For the prediction of secondary structures of c-di-GMP regulatory proteins various online computational tools were used. Expasy SOPMA tool (https://npsa-prabi.ibcp.fr/cgi-bin/npsa_automat.pl?page=/NPSA/npsa_sopma.html) (Geourjon and Deléage, 1995) and GOR IV tool (https://npsa-prabi.ibcp.fr/cgi-bin/npsa_automat.pl?page=/NPSA/npsa_gor4.html) (Garnier et al., 1996) were used to fetch details regarding different secondary structure conformations (percentages of α-helices, β-sheets, turns, extended strands and random coils) of proteins from the given sequences. To identify the various amino acids’ participation in the secondary structure formation, the PSIPRED v.4.0 (http://bioinf.cs.ucl.ac.uk/psipred/) (Buchan and Jones, 2019) server was used.

### 2.10 Tertiary structure prediction and validation

For the comparative analyses of 3D structure of c-di-GMP regulatory DGC proteins, 3D structures were obtained from two of these most advanced professional protein prediction servers AlphaFold database (https://alphafold.ebi.ac.uk/) (Jumper et al., 2021) and TrRosetta (transform-restrained Rosetta) (https://robetta.bakerlab.org/) (Du et al., 2021). The DeepMind AI programme AlphaFold makes predictions about a protein's three-dimensional structure based on its amino acid composition. AlphaFold Database is partnered by DeepMind and EMBL's European Bioinformatics Institute. trROSETTA is a server powered by deep learning and Rosetta for rapid and accurate de novo structure prediction of proteins. For comparative analyses of predicted 3D models of the proteins of AlphaFold and trROSETTA, they were validated through the Ramachandran plot using PROCHECK (Laskowski et al., 1993). To analyse correct stereochemistry of the predicted models furthermore ERRAT (Colovos and Yeates, 1993) server was used. For local and global quality of 3D protein models ModFOLD8 (McGuffin et al., 2021) server was used. Selected models were refined using GalaxyRefine server (Heo et al., 2013). Finally, the refined 3Dmodels were further verified by different model evaluation tools such as protein structure analysis ProSA (Wiederstein and Sippl, 2007) and PSICA (Protein Structural Information Conformity Analysis) webserver (Wang et al., 2019). Additionally, Protein Tools server (https://proteintools.uni-bayreuth.de/) was used to detect salt bridges present in the tertiary structure (Ferruz et al., 2021).

### 2.11 Functional domains and motif analyses

To recognize the functional motifs, present in the retrieved proteins, MEME (Multiple Extraction-Maximization for Motif Elicitation) tool (http://meme-suite.org/) was used (Bailey et al., 2015). MEME Suite is a useful tool for motif analysis, widely used in bioinformatics to identify recurring patterns or motifs in proteins, DNA and RNA. It uses statistical models to find recurring patterns that are generally considered to be biologically significant (Machanick and Bailey, 2011; Bailey et al., 2015). The 'ggmotif' tool embedded in MEME suite enhance the visualization of motifs by extracting and displaying motif information from MEME output files. This package allows integration with phylogenetic data and the creation of sequence logos (Li et al., 2022)

InterProScan server was used to search and predict the functional domains (http://www.ebi.ac.uk/interpro/) of the DGCs and the later were classified accordingly (Zdobnov and Apweiler, 2001). Additionally, Conserved Domain database integrated in the InterProScan server helped to predict the amino acids of the active site of the proteins’ functional domains.

### 2.12 Detection of intrinsically disordered regions

Intrinsically disordered protein regions (IDPRs) are considered to play crucial roles in various biological processes due to their high flexibility and adaptability Lack of a permanent tertiary structure allows them to respond to changes in chemical environment and contribute to very high degree of adaptability (Moses et al., 2020). The dynamic nature of IDPRs makes them difficult to study using traditional structural biology techniques, as they often lack well-defined structures (Chen and Zhang, 2024). In the present study AIUPred (https://aiupred.elte.hu/) (Erdős and Dosztányi, 2024) and flDPnn (https://biomine.cs.vcu.edu/servers/flDPnn/) (Hu et al., 2021) and were used to predict IDPRs.

### 2.13 Protein-protein interaction (PPI)

STRING v.11.5 (http://string.embl.de/) is a database of known and predicted protein interactions (Szklarczyk et al., 2023). It revealed co-expression and protein–protein association of the target c-di-GMP DGCs with other closely associated proteins and generated their subsequent functional networks (Islam et al., 2015).

### 2.14 Molecular docking studies

Template guided blind docking study was performed using CB-DOCK2 (https://cadd.labshare.cn/cb-dock2/) (Liu et al., 2022). Template-based approaches that are computationally less expensive but are capable to provide high accuracy when sufficient structural information are available (Krupa and Krupa, 2024). CB-DOCK2 is the updated version of CB-DOCK with integrated FitDock (Yang et al., 2022). FitDock, is a method that leverages existing structural templates derived from traditional structural biology methods, *viz*., X-ray crystallography NMR spectroscopy, cryo-electron microscopy deposited in the protein data bank (RCSB-PDB) to predict protein-ligand interactions more efficiently and accurately using template guided approach (Liu et al., 2022). CB-DOCK2 outperformed previous blind-docking techniques in terms of success rates for top-ranking poses whose root-mean-squared deviation (RMSD) was within 2 Å from the location in the X-ray crystallography structure. The docked ligand protein interactions were visualised in 2D using Discovery studio software.

### 2.15 Molecular mechanics with generalized born surface area (MM/GBSA) calculations

MM/GBSA uses the Generalized Born model to estimate solvation energies and often used for its computational efficiency (Godschalk et al., 2013). MM/GBSA for each of the protein-ligand docked complex was evaluated using fastDRH (http://cadd.zju.edu.cn/fastdrh/overview) server (Wang et al., 2022) with previously described methodologies (Dutta et al., 2023). Using the Autodock vina engine structure-truncated MM/GBSA energy was calculated along with per-residue energy decomposition predicated on multiple conformations. The receptor-ligand complex derived from CB-DOCK2 served as the reference for the binding pocket. The receptor force field ff19SB (incorporating the OPC water model) and the ligand force field GAFF2 were selected, while the truncation radius parameter was maintained at its default value throughout all rescoring methodologies.

### 2.16 Molecular dynamics simulations

Molecular dynamics simulation was executed on the iMODS tool (https://imods.iqfr.csic.es/) (López-Blanco et al., 2014) to study the stability of protein-ligand docked complex. To assess the reliability and flexibility of the protein-protein complex, normal mode analysis was conducted (Sumera et al., 2022). A 50 ns run was conducted (Santra and Maiti, 2022) for each of the six DGCs docked with its ligand GTP. Generally, 50 ns simulation can provide insights into the stability of a protein-ligand complex, as seen in studies where stable interactions were observed over similar timescales and there are prior studies that used 50 ns simulations for studying ligand-receptor complex of even larger proteins than the studied DGCs, *viz*., P-glycoprotein (Syed et al., 2017), toll like receptor proteins (TLR-7 and TLR-8) (Xiaoyu Wang et al., 2022) acetylcholine esterase (Manandhar et al., 2022). iMODS uses parameters *viz.,* B-factors, deformability, eigenvalues and variance to analyse magnitude of protein-ligand motion (López-Blanco et al., 2014).

## 3 Results and discussion

### 3.1 Compositional heterogeneity based on codon usage

For determination of biasness in codon usage patterns GC compositions have been studied. GC3, which indicates the third position of GC, has greater impact on codon usage patterns. In the studied DGCs it was observed from Table 1. GC content ranged from 44.8 % to 49.5 % and among them *cdgA* possessed the highest GC % (49.5 %) followed by *vpvC* (47.5 %). In case of positions of codons (GC1, GC2, and GC3) in these genes it was observed GC contents ranged from 46.0 % to 55.3 % for GC1, 33.1 %–34.3 % for GC2, and 45.9 %–57.8 % for GC3. Average GC content for DGCs in these three positions were 52.7 % (GC1), 33.7 % (GC2) and 50.0 % (GC3). Occurrence of GC1 % was highest for *cdgA*, *cdgH*, *cdgK* and *cdgM*; whereas GC3 % was highest in case *cdgL* and *vpvC*.

**Table 1:** Different positional nucleotides (GC1 %, GC2 %, GC3 %, GC %, A3 %, T3 %, G3 %, C3 %, and CAI) values of *V. cholerae* DGCs.

Parameters	*cdgA*	*cdgH*	*cdgK*	*cdgL*	*cdgM*	*vpvC*
GC1 %	54.5	55.3	54.1	46.0	54.9	51.5
GC2 %	33.5	33.7	34.3	34.6	33.1	33.2
GC3 %	47.4	47.5	45.9	53.2	48.3	57.8
GC %	49.5	45.5	44.8	44.6	45.5	47.5
A3 %	21.25	20.47	18.57	23.72	20.08	16.94
T3 %	31.34	32.05	35.51	23.12	31.63	25.29
G3 %	24.25	24.63	23.47	19.76	27.65	25.06
C3 %	23.16	22.85	22.45	33.40	20.64	32.71
CAI	0.737	0.751	0.783	0.707	0.783	0.723
Nc	49.5	55.2	49.9	53.0	53.3	52.5

Moreover, the frequencies of four nucleotides on the third position of codons were as follows: Usage of (U/T) 3 % was observed to be higher for *cdgA*, *cdgH*, *cdgK* and *cdgM* suggesting a bias towards A or U/T at third codon positions (Ling et al., 2024). Similarly, the codon usage bias in *Chlamydia trachomatis* serovars, which prefer A/U ending codons, suggests that understanding these patterns could aid in identifying drug targets and understanding pathogen evolution (Sadhasivam and Vetrivel, 2018). In contrast, C3 % were higher for *cdgL* and *vpvC* indicating GC bias at the third position of codon. Codon bias plays crucial roles in different cellular and molecular events, *viz.*, transcription, mRNA stability, translation efficacy, *etc.* (Ling et al., 2024; Quax et al., 2015). Reports reveal that GC3 % is considered a key factor in shaping amino acid compositions; with GC-rich codons contributing to higher evolutionary rates in genes from the last universal common ancestor (Du et al., 2018). The present findings indicate among studied DGCs natural selection was exhibited higher potential shaping codon usage of *cdgL* and *vpvC* than the other DGCs as they had higher GC % (>50 %) in the third position of codons.

### 3.2 Effective number of codon (ENc) plot analyses

**Fig. 1:**
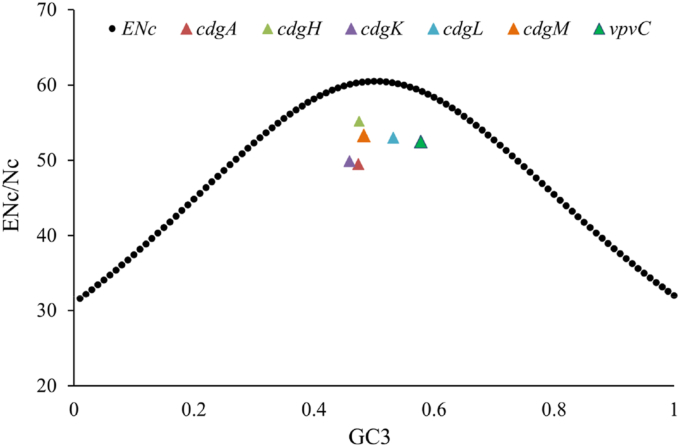
**ENc plots showing the relationship between ENc values and GC3s of *V. cholerae* DGCs:** The solid black line reflects the predicted curve (ENc). All studied DGCs were found below the predicted curve indicating low mutation pressure on shaping the codon usage patterns.

The ENc plot, an efficient technique in assessing the codon usage patterns, was employed to investigate the influence of GC3s on codon usage bias. Nc values of *V. cholerae* DGCs were in the range 49.5–55.2 (Table 1). Nc value > 45 demonstrates weak codon bias (Chaudhary et al., 2023), thereby depicting low codon usage bias in the studied DGCs. Low codon usage bias corresponds to usage of multiple codons for each amino acid, allowing gene expressions more efficiently in the different environments (Chen et al., 2014). From the plot of the Nc values it was evidenced that all the studied DGCs were below the predicted ENc curve (Fig. 1). The Nc values appearing at or above the predicted curve suggest selectional bias due to mutational pressure whereas Nc values below the expected curve and appear near to the curve indicate mutational pressure was not the primary contributor (Dey et al., 2022). Thus, it could be concluded that in the studied DGCs mutational pressure played minimal role in shaping the codon usage patterns. Beside other forces, *viz*., natural selection, gene length, tRNA abundance, or RNA structure, synergistically contribute to the said phenomenon (Yu et al., 2021).

### 3.3 Neutrality plot and parity rule 2 (PR2) bias plot analyses

**Fig. 2:**
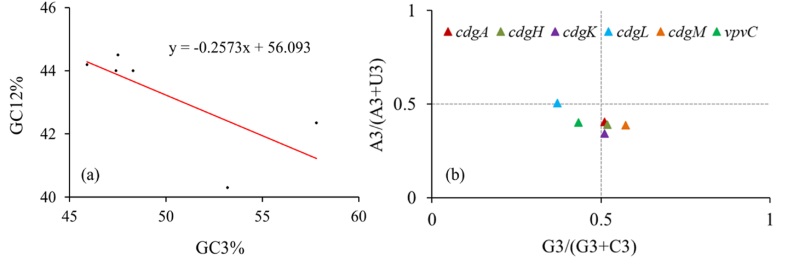
**Sources of codon usage in *V. cholerae* DGCs:** (a) Neutrality analysis performed by plotting GC12s values against GC3s values of six DGCs. The regression line is represented by the red straight line. GC12 and GC3 values were found to have negative correlation suggesting natural selection to be the major responsible factor (74.27 %) in shaping codon usage patterns. (b) Parity rule 2 plot analyses revealed for most of DGCs third base of codon is inclined towards U over A and G over C in AU ending and GC ending codons respectively.

To further explore the sources of codon usage in *V. cholerae* DGCs neutrality and parity rule 2 (PR2) bias plots were analysed. Neutrality plot analysis is related to the role of natural selection in addition to mutational pressure on codon usage patterns. In neutrality plot, when the slope is near about one it indicates that the codon usage is influenced by mutation. Values close to zero demonstrates natural selection is the key driving force for shaping the codon usage (Ling et al., 2024; Yu et al., 2021). The calculated slope was −0.2573 for DGC genes (Fig. 2a) suggesting natural selection played key role (contributing 74.27 %) in shaping the codon usage patterns in case of the studied DGCs of *V. cholerae*. It was also found that the GC12s and GC3s values were inversely correlated (r = −0.706; P < 0.05).

To further evaluate the impact of mutation and selection pressure on the third codon, PR2 plot was taken into consideration. DGCs were unevenly distributed in three quadrants distant from the central region of 0.5, which is the equilibrium state (A = T/U; C ≡ G) (Yu et al., 2021). Presence of genes near this equilibrium point indicate codon usage preference is entirely contributed by mutation and distant from this point suggesting natural selection played stronger role in codon usage (Ling et al., 2024). Among the *V. cholerae* DGCs natural selection was predominant in selection of codon preferences as established by the non-residence of the DGC genes near the equilibrium line and *cdgL* and *cdgK* had highest biasness towards AT and GC ending codons respectively (Fig. 2b). Except *cdgL* for all other most DGCs *viz., cdgA*, *cdgH*, *cdgK*, *cdgM* and *vpvC* A3 %/(A3 %+T3 %) ratio were below 0.5 suggesting frequency of codon base usage, specifically the third base of codon is inclined towardsT(U) over A. While G3 %/(G3 %+C3 %) ratio were higher than 0.5 in majority of *V. cholerae* DGCs (except *cdgL* and *vpvC*) indicating in GC ending codons third base lean towards G over C (Ling et al., 2024).

### 3.4 Relative synonymous codon usage (RSCU)

RSCU represents a computationally derived metric that assesses the utilization of different codons corresponding to a specific amino acid within a protein. It is helpful in elucidating the codon usage patterns of diverse proteins across multiple organisms (Dey et al., 2022). Table S1 (supplementary section) provides a comprehensive overview of the RSCU values for all DGCs. Majority of the DGCs exhibited preference for codons terminating in AU. Interestingly, in case of Leu (the most abundant amino acids in the studied DGCs) GC ending codon (UUG) was significantly (*P* < 0.05) preferred over AU ending codons but second most abundant amino acid Ile exhibited significant (*P* < 0.05) biasness towards AU ending codons (AUU). Asp which is the key amino acid for performing nucleophilic attack on GTP to facilitate its binding (Guo et al., 2017) showed biasness towards AU ending codon in all six studied DGCs. The inclination towards specific codons was further evaluated (Table S1; supplementary section) in relation to RSCU metrics. For a codon with mean RSCU value > 1.6 is considered over-represented and mean RSCU value < 0.6 denotes under-representation (Yu et al., 2021). Current observations reveal only one codon AGT (encodes Ser) was significantly over-represented (*P* < 0.05) and significant underrepresentation (*P* < 0.05) was observed for seven codons *viz*., CTA (encodes Leu), ATA (encodes Ile), TCT (encodes Ser), TCA (encodes Ser), CCT (encodes Pro), GCA (encodes Ala), AAG (encodes Lys) across all studied DGCs of *V. cholerae*. Overall, such understanding and leveraging on codon usage patterns can significantly enhance the design and development of drugs, particularly in the context of recombinant protein production and pathogen-targeted therapies (Diambra, 2017).

### 3.5 Codon adaptation index (CAI)

The expected transcription levels of six diguanylate cyclase (DGC) genes in *V. cholerae* can be inferred by calculating the CAI vlaues. As represented in Table 1, CAI values ranged from 0.707 (CdgL) to 0.783 (CdgM and CdgK), thereby suggesting a significantly elevated expression of genes characterized by a pronounced codon adaptation. Variability in codon bias encompasses biased codon pairings alongside codon interrelations. While the initiation of gene expression constitutes a critical phase in the biosynthesis of proteins, codon bias is implicated in a different cellular process, particularly variations in the production levels and folding (Quax et al., 2015). CAI value above 0.6 is generally considered indicative of strong adaptation and potential high expression levels (Brandão, 2012). All DGCs demonstrate CAI values that exceed 0.6, which implies an elevated degree of codon adaptation and display high expression pattern in *V. cholerae* in various environmental conditions. Use of codon adaptation index (CAI) in conjunction with subtractive genomics has been proposed as one of the effective methods in identifying potential drug targets in bacterial pathogens, highlighting the utility of codon usage analysis in drug discovery (Vetrivel et al., 2011).

### 3.6 Subcellular localization and topological analysis

Three different tools (PSORTdb V.4.0, Gneg-PLoc server, and LocTree3) were used to find out subcellular localization of c-di-GMP regulatory proteins. All the servers predicted CdgA, CdgH, CdgL, CdgM, VpvC to be present in the *V. cholerae* cellular membrane. However, PSORTb v.4.0 server predicted CdgK could be localized either in the cell membrane or cytoplasm but all other tools predicted CdgK to be localized in the cell membrane (Table 2).

**Table 2:** Subcellular localization c-di-GMP regulating DGCs in cytoplasm (C) and/or cell membrane (M) as predicted by bioinformatic tools.

Tools	CdgA	CdgH	CdgK	CdgL	CdgM	VpvC
**PSORTdb V.4.0**	M	M	C/M	M	M	M
**Gneg-PLoc**	M	M	M	M	M	M
**LocTree3**	M	M	M	M	M	M

Studies reveal that among the GGDEF containing DGCs in *Pseudomonas aeruginosa* six are present in cell membrane(Valentini and Filloux, 2016). DGCs having transmembrane regions are also found in *Salmonella typhimurium*, *Pseudomonas aeruginosa* and *Escherichia coli* (Simm et al., 2004).

Comparative topological analyses of c-di-GMP regulatory DGC proteins using Deep TMHMM, MEMSAT 2, TOPCON, CCTOP, Phobius, TMSEG online servers analysed the details of transmembrane (TM) regions in all the proteins. The transmembrane regions predicted by various servers are presented in Table 3.

**Table 3:** Topological analyses of c-di-GMP regulating DGCs indicating the transmembrane domains along with its stretches in protein chain using different computational servers.

Proteins	No of predicted TM helices	Predicted AA residues in TM region					
		Deep TMHMM	MEMSAT 2	TOPCON	CCTOP	Phobius	TMSEG
**CdgA**	2	17–35	15–35	15–35	12–35	12–35	16–35
		180–199	181–200	180–200	183–200	181–200	183–196
**CdgH**	1	513–529	512–529	511–531	513–532	513–532	511–524
**CdgK**	2/1	9–26	17–32	8–28	7–25	303–322	7–27
		302–322	303–322	303–322	305–322		301–322
**CdgL**	2	13–30	15–30	10–30	10–30	6–28	16–33
		261–281	261–289	261–281	261–283	261–283	262–275
**CdgM**	2/1	17–37	22–39	19–39	17–36	315–335	17–39
		315–335	318–334	315–335	315–334		315–336
**VpvC**	2/1	17–34	148–172	14–34	17–34	14–34	14–38
		154–172		151–171	151–172	150–152	154–177

**Fig. 3:**
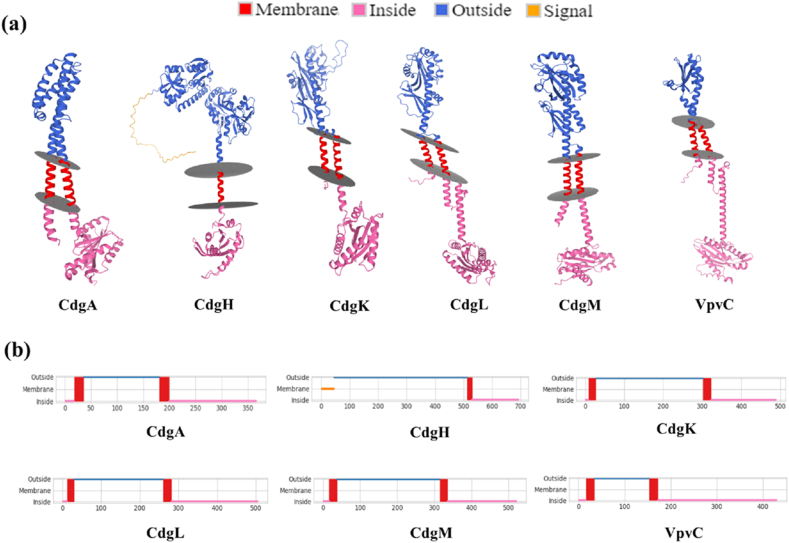
**Structure of transmembrane domains of c-di-GMP regulatory DGCs:** (a) 3D visualization of transmembrane regions of the c-di-GMP regulatory DGCs were performed using MembraneFold server. (b) The membrane spanning regions of individual proteins in respect to their amino acids sequences were visualised using Deep TMHMM server.

CdgA and CdgL through all the specified servers predicted to have two TM regions, whereas presence of a single TM region was detected in CdgH by all the specified servers. Except the Phobius, all other tools predicted the occurrence of 2 TM helices in CdgK and CdgM. In case of VpvC MEMSAT2 predicted single spanning TM region but other five servers predicted two TM regions. These differences might be due to the fact that TM domains of CdgK, CdgM and VpvC do not meet the cutoff of that specific tool(Kaur et al., 2020). The TM regions could be specified and visualised in Fig. 3. Additionally, presence of signal peptide was detected using Deep TMHMM and Signal P v6.0 web tool. Presence of signal peptide was detected only in CdgH among studied DGCs by both the tools. Signal P server predicted the signal peptide cleavage site to be present between Ala45 and Ala46 amino acid residues for CdgH.

### 3.7 Crystallization analyses

Expert pool (EP) and random forest (RF) are the two crystallization classification techniques used by XtalPred; several protein features (length, isoelectric point, gravy index, predicted structural disorder, instability index, predicted coil secondary structure, predicted coiled-coil structure, and insertion score) for which separate crystallization probabilities that have been calculated are combined into a single crystallization score in the EP method. Score ranges from 1 to 5. Lower the score higher is the crystallization probability of the protein (Slabinski et al., 2007). For all the DGCs it was observed that EP crystallizability score was 5 (Fig. S1; Supplementary Section) signifying that they were very tough to crystalize.

On the other hand, the second type of crystallization classification technique named RF classifier utilizes some other protein features, *viz.*, amino-acid composition of the predicted protein surface, predicted surface ruggedness, hydrophobicity, and side-chain entropy of surface residues (Jahandideh et al., 2014). In case of the DGCs CdgL exhibited the lowest random forest crystallization score of 6 and the highest score of 11 in case of CdgH and VpvC. Score for each of CdgK and CdgM was 10 and CdgA scored 9 in this technique (Fig. S1; Supplementary Section). Finally combined class score predicted by Xtalpred server using EP and RF technique for all the DGCs was 5. Similar result was observed for the outer membrane protein Omp33-36 in *Acinetobacter baumannii* (Jahangiri et al., 2018). This combined class score ranged between 1 and 5, where 1 indicating the protein is very likely to get crystalized and a score of 5 indicates the protein is less likely to get crystalized. Thus, it could be interpreted that it is very hard to crystalize the studied DGCs so *in silico* structural study may be considered as a good alternative for their structural evaluation.

### 3.8 Physicochemical properties

Physicochemical properties, *viz.*, protein length (aa), molecular weight, isoelectric point (pI), total number of negatively and positively charged amino residues, extinction coefficient, instability index (II), aliphatic index (AI), and grand average of hydropathicity (GRAVY) of c-di-GMP regulatory DGCs were calculated using the ExPASy ProtParam tool. The results are summarized in Table 4.

**Table 4:** Physico-chemical properties of c-di-GMP regulatory diguanlylate cyclases (DGCs).

Cyclic di GMP regulatory DGCs	NCBI locus tag	No. of AAs	MW	pI	(−)R	(+)R	ε	II	AI	GRAVY
CdgA	VCA0074	366	42031.56	6.56	36	34	27515	38.34	96.78	−0.057
CdgH	VC1067	693	79308.94	6.81	79	77	88380	39.47	99.19	−0.169
CdgK	VC1104	489	55593.61	5.17	63	44	60990	41.58	101.10	−0.043
CdgL	VC2285	505	57237.35	5.32	62	46	61685	42.98	94.61	−0.039
CdgM	VC1376	521	60285.17	5.39	75	60	62925	42.85	101.71	−0.159
VpvC	VC2454	430	48793.08	5.67	56	45	44475	38.99	111.84	−0.079

MW = molecular weight; pI = isoelectric point; (−) R = total number of negatively charged residues; (+) R = total number of positively charged residues; ε = extinction coefficient (a units of M^−1^ cm^−1^ at 280 nm measuring in water); II = instability index; AI = aliphatic index; GRAVY = grand average of hydropathicity.

Among the DGCs, CdgH is the largest with highest amino acids chain length (693 aa) and highest molecular weight (79.3 kDa) while CdgA is the smallest protein with smallest amino acids chain length (366 aa) and lowest molecular weight (42.03 kDa).

The isoelectric point (pI) is the pH value at which a molecule effectively carries no electrical charge (zwitterionic state) that causes its zero electrophoretic mobility. Additionally, proteins become stable and compact at its isoelectric pH, therefore calculated pI values are considered be useful in preparing a buffer system for isoelectric focusing technique purification (Dey et al., 2022). The theoretical pI derived from ProtParam revealed all the proteins to be acidic in nature, CdgK (pI 5.17) and CdgH (pI 6.81) being the most and least acidic, respectively. Similar results were obtained from Antarctic *Rhodococcus* sp. NJ-530 which had acidic DGC of 34.6 KDa with pI of 5.58 (Wang et al., 2022). Solubility, molecular interactions, and subcellular localization of protein are influenced by the isoelectric point and the quantity of positively and negatively charged residues (Kaur et al., 2020).

Computational studies using Expasy's Protparam reveal that at 280 nm, the extinction coefficient (ε) of the DGC proteins ranges from 27515 M^−1^cm^−1^ to 88380 M^−1^cm^−1^. Tryptophan, tyrosine, and cysteine residues per molecule are used to determine a protein's EC value because they considerably affect the optical density of protein measured in the wavelength range of 276–282 nm (Islam et al., 2015). CdgH and CdgK were found to be tyrosine rich among the DGCs. CdgH showed highest EC due to presence of highest no of tryptophan (37) and tyrosine (6) among the DGCs studied herewith. CdgA had the least amount of tryptophan (2), tyrosine (11), and cysteine (2). This calculated EC can be used to analyse protein-protein and protein-ligand interactions quantitatively in solutions (Islam et al., 2015).

Instability index (II) is calculated based on the dipeptide composition of a protein. It provides an estimate of the protein's stability *in vitro* by evaluating the likelihood of degradation or denaturation under laboratory conditions (Gamage et al., 2019). It is used to predict the stability of proteins when designing mutants or optimizing proteins for industrial applications. It helps in identifying potentially unstable regions that may require modification to enhance stability (Norrild et al., 2022). II of CdgA, CdgH, VpvC were found to be lower than 40 suggesting that they are stable in test tube conditions. But CdgK, CdgL, and CdgM were found to have high levels of instability which indicated that performing structural studies on these proteins could be challenging as they will be less stable (due to II > 40) in *in vitro* conditions (Islam et al., 2015; Dey et al., 2022). Hence bioinformatics-based approaches could be considered as a viable alternative for studying their structural properties.

Aliphatic index (AI) is another metric to assess the protein stability in addition to instability index (II). The proportion volume occupied by the aliphatic side chains of amino acids like alanine, valine, leucine, and isoleucine in a protein is known as AI. In contrast to proteins with lower AI values, (which are not thermally stable but exhibit more flexibility), proteins with very high AI may exhibit stability throughout a large temperature range (Islam et al., 2015). AI of the studied DGCs ranged between 94.61 and 111.84. So, it could be suggested that all the studied c-di-GMP regulatory DGCs are thermostable (Dey et al., 2022).

Moreover, GRAVY value for a protein corresponds to the hydropathy values of all the amino acids (Ikai, 1980). In the present study, GRAVY score of the DGCs had a range from −0.039 to −0.169. According to previous studies, a protein's positive GRAVY value suggests that it is hydrophobic, whereas a negative GRAVY value indicates its hydrophilic nature (Chang and Yang, 2013). In respect to that all studied DGCs appear to be hydrophilic in nature. Lower the GRAVY value higher is its ability of interaction with water. Results indicate CdgL and CdgK have lower interaction abilities with water whereas CdgH and CdgM have higher interaction abilities.

**Fig. 4:**
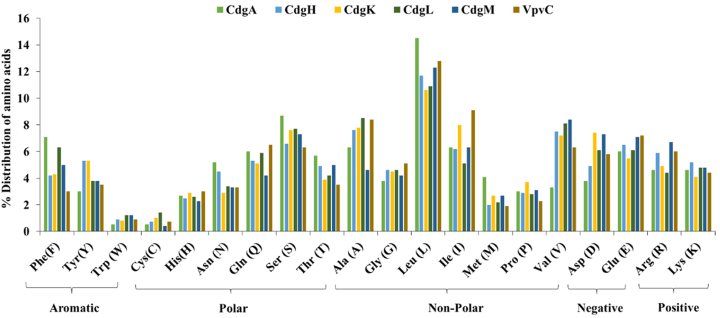
**Amino acid composition with respect to their percentage of occurrence in the studied DGCs:** Leucine (L) was found to be the most predominant amino acid closely followed up by isoleucine (I) and valine (V). Tryptophan (W) and Cysteine (C) were the least abundant amino acids in DGCs.

Amino acid composition of c-di-GMP regulators, with respect to aromatic, polar, non-polar, positively and negatively charged amino acids are shown in Fig. 4. Leucine was found to be the most abundant amino acid to be present in all the DGCs. CdgA contained highest amount of leucine (14.5 %) among the DGCs studied. Other abundant non-polar aliphatic amino acids in all DGCs were found to be isoleucine and valine but interestingly CdgA contained very less amount of valine (3.3 %). In case of non-polar aromatic amino acids tryptophan was found to be present in very low amount but tyrosine and phenylalanine were adequately distributed among CdgA, CdgH, CdgK, CdgL, CdgM but VpvC lacked aromatic amino acids among the DGCs. Non-polar acids help to stabilize the tertiary structure by hydrophobic interactions (Dyson et al., 2006). Studies revealed that tyrosine is important to stabilize the aqueous interface of trans-membrane proteins (Stillwell, 2016). Among polar uncharged amino acids Serine (8.6 %) was most abundant and found in highest amount in CdgA whereas, cysteine was the least abundant amino acid, and its lowest presence was detected in CdgM (0.4 %). These uncharged polar amino acids help to stabilize the protein by formation of hydrogen bonds (Vijayakumar et al., 1999). In case of polar charged amino acids, the negatively charged Aspartic acid and Glutamic acid predominated over the positively charged ones. These charged amino acid residues are involved in salt bridge formation to stabilize tertiary protein structures (Donald et al., 2011).

### 3.9 Secondary structure evaluation

**Fig. 5:**
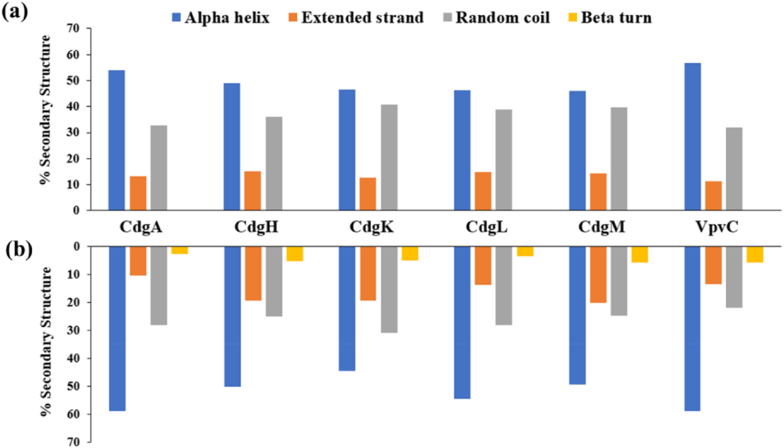
**Percentage occurrence of secondary structural features in DGCs:** (a) GOR4 and (b) SOPMA servers were used to predict the secondary structures. Both servers predicted alpha helix was the predominant secondary structural feature present in the studied proteins. Interestingly SOPMA server predicted presence of Beta turns in the DGCs which were absent in the analysis of GOR4.

Computational tools *viz.,* GOR4 and SOPMA were used to predict percentage occurrence of secondary structure features (α-helices, extended strands, β-turns and random coils) of c-di-GMP regulatory DGCs (Fig. 5). Interestingly, it was found that GOR4 predicted complete absence of β–sheet for all the DGCs, but SOPMA analysis revealed presence of very less amount of β-sheets (<6 %) in all the studied proteins. Structural investigations of diguanylate cyclase PleD of *Caulobacter crescentus* revealed the catalytic GGDEF domain forms a five-stranded β-sheet encircled by helices similar to the catalytic centre of adenylate cyclase (Chan et al., 2004). Presence of β-sheets in *V. cholerae* DGCs by SOPMA indicate similar catalytic domain might be present in the cholera pathogen. α-helix was found to be predominant in all the DGCs. Presence of α-helix in high amount is a common feature for TM proteins (Popot, 1993). Among the studied DGCs VpvC had maximum percentage of α-helix in its secondary structure according to both GOR4 (56.74 %) and SOPMA (58.84 %). GOR4 predicted CdgM had lowest α-helix distribution (46.07 %) but according to SOPMA CdgK had the lowest α-helix distribution (44.58 %) among the studied proteins. Random coil was the second highest type of secondary feature found in all the proteins (>31 % & >21 % in GOR4 and SOPMA respectively). The coils are useful for linking two TM regions of the proteins (Tastan et al., 2009). To check participation of each amino acid in secondary structure formation PSIPRED server was used. The results are displayed in Fig. S2. (Supplementary section) PSIPRED also predicted presence of Beta strands in the said proteins.

### 3.10 Tertiary structure prediction and validation

Membrane proteins are present in low abundance in their natural environments, necessitating its overexpression in heterologous systems. This can lead to issues with protein folding and stability, complicating structural studies (Granseth et al., 2007; Pandey et al., 2016). The *ab initio* modelling, which offers plausible predictions of a protein's structure, is based on the existing protein models in the Protein Data Bank (PDB) database for machine/deep learning. With the recent advancements of structural bioinformatics, numerous online servers are available to be used to predict protein tertiary structures. To compare the accuracy of the computational servers in every two years, a community-wide experiment called Critical Assessment of Structure Prediction (CASP) for protein structure prediction is conducted. AlphaFold and its updated AlphaFold 2 servers won CASP13 and CASP14 in 2018 and 2020 respectively (Heaven, 2020; Sample, 2018). However, in 2022 trRosetta server (also known as 'Yang-Server') ranked on the top at CASP15 (Valdés-Tresanco et al., 2023). In this study the predicted 3D structures of the c-di-GMP regulatory DGCs were fetched from AlphaFold database and trRosetta server for further studies. It is well established that both AlphaFold2 and RosettaFold are capable of predicting protein structures with very high accuracy despite substantial architectural differences and both are able to generate accurate models protein complexes besides protein monomers (Baek et al., 2024). There are prior studies that relied on protein structures predicted by AlphaFold for drug discovery studies, *viz*., design selective inhibitors for HDAC11, a histone deacetylase enzyme (Baselious et al., 2024) against neuroblastoma, discovery of psychotropic agonists targeting the trace amine–associated receptor 1 (TAAR1) (Díaz-Holguín et al., 2024). The precision demonstrated by AlphaFold2 in predicting three-dimensional conformation of proteins has made protein targets more accessible to the drug screening (Borkakoti and Thornton, 2023).Similarly, trRosetta is particularly noted for its rapid and accurate de novo structure prediction, making it a valuable tool for generating initial models for docking studies (Du et al., 2021). Rosetta has been extensively used in protein-protein and protein-small molecule docking, employing flexible backbone refinement to improve model accuracy (Burman et al., 2019). trRosetta-predicted structures integrated into AlloMAPS 2 database provide insights into allosteric effects of mutations and small probe binding. These information are crucial for fragment-based design of allosteric effectors, which are potential drug candidates (Tan et al., 2023).An interesting study further reports Rosetta, when combined with AlphaFold ensembles, can enhance the prediction of enzyme thermostability. This approach is more accurate than using crystallographic structures alone, as it reduces scaffold bias and provides robust predictions of thermostability trends (Peccati et al., 2023). Prior report also reveals that AlphaFold predicted structural models could successfully predict ligand binding poses (RMSD <2 Å) and are thus very useful while considering the virtual screening approaches (Alhumaid and Tawfik, 2024) Models from both the servers were compared for each DGCs using three online tools *viz.,* PROCHECK, ERRAT and ModFOLD8. PROCHECK looks for the energetically allowed regions for backbone dihedral angles ψ against φ of the amino acid present in protein structure using Ramachandran plot (Laskowski et al., 1993). ERRAT measures overall quality factor of the model by analysing the statistics of non-bonded interactions between different atoms (Colovos and Yeates, 1993). ModFOLD8 server generates precise estimations of the local and global quality of 3D protein models. Utilising neural networks, it integrates the inputs from thirteen distinct scoring techniques (McGuffin et al., 2021). It was found that structures predicted from both the servers were satisfactory and usable for further studies. However, in this study the predicted structure of each DGC that scored higher in two out of three severs (PROCHECK, ERRAT and ModFOLD8) was selected for further analysis. The detailed results of these analyses are represented in Table 5.

**Table 5:** Comparative analysis of predicted DGCs structures between AlphaFold DB and trRosetta server.

DGCs	PROCHECK (% Residues in most favoured region in Ramachandran Plot)		ERRAT (Overall Quality Factor of predicted model)		ModFOLD8 (Global model quality score)	
	AlphaFold	trRosetta	AlphaFold	trRosetta	AlphaFold	trRosetta
CdgA	**96.50**	95.30	**98.87**	97.16	0.50	**0.51**
CdgH	88.10	**93.90**	94.59	**97.75**	**0.48**	0.46
CdgK	**93.30**	92.20	95.40	**97.07**	0.41	**0.42**
CdgL	**94.00**	91.40	**96.95**	79.48	**0.44**	0.42
CdgM	**95.00**	94.20	**97.16**	97.04	0.43	**0.44**
VpvC	94.70	**96.00**	**98.01**	94.63	**0.47**	0.46

Bold numerical correspond to high scores predicted by structure validation tools.

**Fig. 6:**
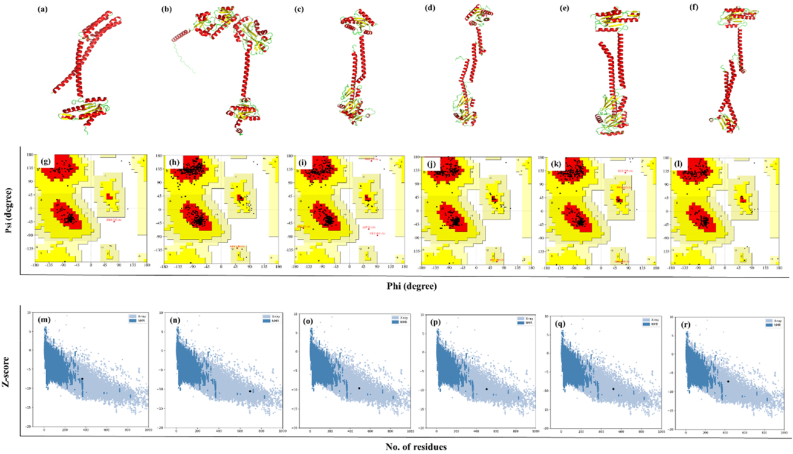
**3D models of DGC proteins and their evaluations:** Structures of CdgA, CdgH, CdgK, CdgL, CdgM and VpvC proteins, respectively (a, b, c, d, e, f); Ramachandran plots of CdgA, CdgH, CdgK, CdgL, CdgM and VpvC showing amino acid placement in allowed and disallowed regions (g, h, i, j, k, l); Z-scores of CdgA, CdgH, CdgK, CdgL, CdgM and VpvC proteins evaluated using ProSA (m, n, o, p, q, r).

It was found that the AlphaFold structure were much reliable for CdgA (scored higher in PROCHECK and ERRAT), CdgL (scored higher in PROCHECK, ERRAT and ModFOLD8), CdgM (scored higher in PROCHECK and ERRAT), and VpvC (scored higher in ERRAT and ModFOLD8) whereas trRosetta better predicted the structures of CdgH (scored higher in PROCHECK and ERRAT) and CdgK (scored higher in ERRAT and ModFOLD8). Hence these structures were selected, and further refinement of the structures were carried out using GalaxyRefine server which performs side chain rebuilding and repacking followed by molecular dynamics simulation for relaxation of the overall structure. Final refined structures could be visualised and presented in Fig. 6(a–f). PROCHECK revealed improved Ramachandran Plot for the DGCs (Table 6) after refinement.

**Table 6:** Ramachandran plot statistics of final refined 3D models of *V. cholerae* selected DGC proteins using PROCHECK.

Amino acid residues (%)	CdgA	CdgH	CdgK	CdgL	CdgM	VpvC
Most favoured regions	98.8	96.4	95.7	96.1	97.9	98.0
Additionally allowed regions	1.2	3.4	3.4	3.6	1.7	2.0
Generously allowed regions	0.0	0.2	0.2	0.2	0.2	0.0
Disallowed regions	0.0	0	0.7	0.0	0.2	0.0

Amino acid residues participated in most favoured regions for CdgA, CdgL, CdgM, and VpvC were 98.8 %, 96.4 %, 95.7 %, 96.1 %, 97.9 %, and 98 %, respectively (Fig. 6g-l). Post refinement quality of refined models were further assessed by ProSA server. It measures the overall protein structure by assessment through Z-plot (Wiederstein and Sippl, 2007). Z-score for the predicted structures of CdgA, CdgL, CdgM, and VpvC were −7.48, −10.56, −9.64, −9.75, −9.51 and −7.26, respectively. Z-score of predicted 3D structure of CdgA was located in the overlapping space of X-ray and NMR protein structures (Fig. 6m). While the rest other DGCs were in the space of X-ray crystallography protein structure (Fig. 6n-r). To identify any misfolding in the protein structure PSICA webserver was used (Wang et al., 2019). It is the official implementation of MUfoldQA (Zhang et al., 2011). It predicted protein misfolding by a global score which is an estimation of GDT-TS score and ranged between 0 and 1. The estimated global scores for the predicted model of *V. cholerae* DGCs, *viz*., CdgA, CdgH, CdgK, CdgL, CdgM and VpvC were 0.504, 0.4667, 0.4064, 0.4464, 0.4304 and 0.5078, respectively. Global score below 0.2 indicates serious misfolding in the predicted model and score higher than 0.5 indicated predicted model had similar structure as its original tertiary structure. The results indicated that no misfolding was present in the predicted models and the predicted model for CdgA and VpvC were very close to its original structure.

**Fig. 7:**
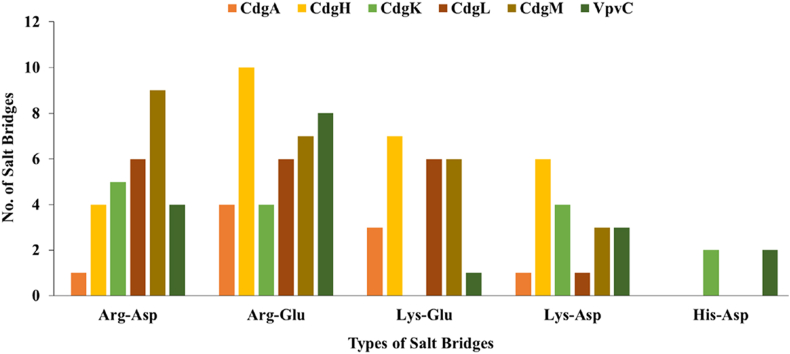
**Types of salt bridge present in DGCs:** Arg-Asp and Arg-Glu salt bridge was mostly found in DGCs and might be responsible for stabilization of tertiary structure. His-Asp salt.

Salt bridges are very important in stabilizing the protein tertiary structures. Different types of salt bridges in the studied DGCs were detected using ProteinTools server. It was predicted that in all the six DGCs arginine was the most abundant amino acid to participate in salt bridge formation and histidine was least involved in salt bridge formation. In CdgA, CdgH and VpvC the Arg-Glu salt bridge was most common whereas Arg-Asp salt bridge was predominant in CdgK and CdgM. Interestingly, CdgL had equal number of Arg-Glu, Arg-Asp and Lys-Glu salt bridges (Fig. 7). When polar amino acids are exposed to a hydrophobic environment in membrane proteins, salt bridges lead to have a more pronounced impact. Charged residues in TM helices prevent proper membrane insertion. Energy cost of membrane insertion is reduced by formation of salt bridges among the charged residues (Duart et al., 2022) To conclude, it may be interpreted that all predicted structures of the DGCs were of good quality and could be of use in study of bacterial cellular processes.

### 3.11 Domain and motif analyses

InterPro server is useful to determine the domain positions in proteins. In the present study it was observed that all the DGCs possess GGDEF domain that belong to overlapping homologous superfamilies of nucleotide cyclases (Fig. S3; Supplementary section). Signal transduction is facilitated by the GGDEF domain, which is also believed to catalyse the formation of cyclic di GMP (Ausmees et al., 2001). CdgA was found to possess GGDEF domain at the stretch of 200–366 aa residues. Observations on CdgH reveal presence of a total of three domains, first two domains range from 50th −251^th^ aa & 270^th^ −517^th^ aa residues respectively belong to solute-binding protein family 3/N-terminal domain of MltF. These are components of bacterial ABC (ATP-binding cassette) importers, which are active solute transporters across the cytoplasmic membrane (Singh and Röhm, 2008). This study also reveal CdgH has the GGDEF domain over the stretch of 522^nd^ −686^th^ aa residues. GGDEF domain of CdgK was present at the stretch of 324–489 aa residues. CdgL constituted of two functional domains, first CHASE4 domain that is located in the region from 58^th^ -212^th^ aa residues. In several kinds of transmembrane receptors CHASE4 is present and acts as an extracellular sensory domain. Particularly this domain is present in putative diguanylate cyclases/phosphodiesterases in bacteria and histidine kinases in archaea (Zhulin et al., 2003). Secondly, the GGDEF domain was found to be present at the region of 328^th^ −504^th^ aa residues. CdgM appears to have CHASE domain at 81^st^ −302^nd^ aa residues. Always found N-terminally in extracellular or periplasmic regions the CHASE domain is projected to facilitate signal transduction, by binding to a variety of low-molecular-weight ligands, including cytokinin-like adenine derivatives or peptides. This domain is made up with two extended α-helices on both boundaries and two central alpha helices separated by β-sheets (Mougel and Zhulin, 2001) and the GGDEF domain was present in region of 349^th^ −518^th^ aa. In this study *V. cholerae* CdgM was found to possess highest amount of β-sheets (Fig. 3b) among all the studied DGCs by SOPMA analysis. This may be due to occurrence of β-sheets in both GGDEF and CHASE domains. In case of VpvC, interestingly HAMP domain and GGDEF domains were found from 174^th^ −228^th^ & 230^th^ −411^th^ aa residues respectively. HAMP domain, present in bacterial sensor and chemotaxis proteins functions along with the GGDEF domain. By inducing conformational changes in periplasmic ligand-binding domains to cytoplasmic signalling kinase and methyl-acceptor domains, the HAMP domain controls the phosphorylation or methylation of homodimeric receptors (Hulko et al., 2006). The GGDEF domain regulates various cellular functions *viz.,* repression of motility, from motile-to-sessile life transition, production of biofilm matrix, virulence repression by increased synthesis of c-di-GMP (Conner et al., 2017). Additionally conserved domain (CDD) integrated in the InterPro Server helped to predict the active site amino acid residues of the DGCs. Active site residues are demonstrated in Table 7.

**Table 7:** Active site amino acid residues of c-di-GMP regulatory DGCs predicted by InterPro Scan.

DGCs	Predicted active site residues
**CdgA**	Lys249, Asn252, Asp253, His257, Asp261, Arg284, Gly286, Gly287, Asp288, Glu289
**CdgH**	Lys570, Asn573, Asp574, His578, Asp582, Arg602, Gly604, Gly605, Asp606, Glu607
**CdgK**	Lys373, Asn376, Asp377, His381, Asp385, Arg407, Gly409, Gly410, Glu411, Glu412
**CdgL**	Lys380, 383Asn, Asp384, His388, Asp392, Arg415, Gly417, Gly418, Glu419, Glu420
**CdgM**	Lys399, Asn402, Asp403, His407, Asp411, Arg433, Gly435, Gly436, Asp437, Glu438
**VpvC**	Lys278, Asn290, Asp291, His295, Asp299, Arg324, Gly326, Gly327, Asp328, Glu329

**Fig. 8:**
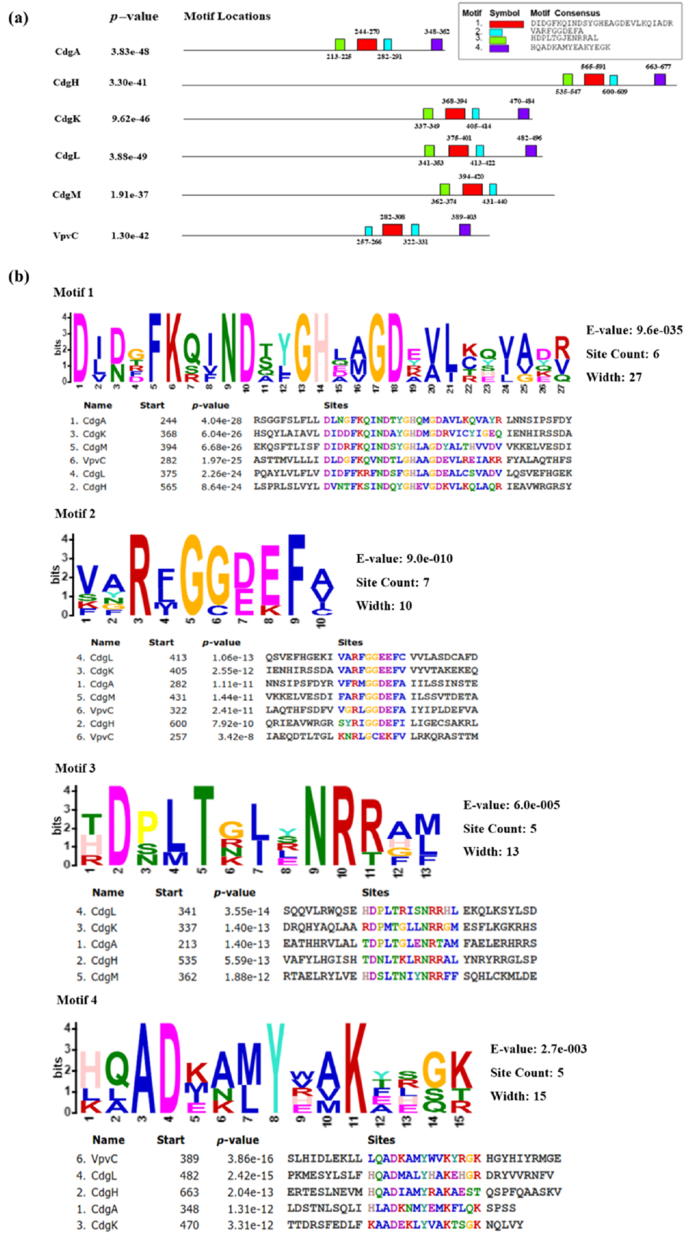
**Analyses of motifs in DGCs** (a) Distribution of conserved motifs and (b) their multiple sequence alignments of the motifs present in amino acid sequences of the proteins.

Amino acid sequences of DGCs were used as input sequences in the MEME suite to predict the location of motifs in the protein structures. Four types of motifs with the consensus sequences DIDGFKQINDSYGHEAGDEVLKQIADR, VARFGGDEFA, HDPLCTGJENRRAL, and HQADKAMYEAKYEGK were abundant among the DGCs (Fig. 8a). It was observed among them CdgA, CdgH, CdgK, CdgL possessed four types of motifs. CdgM had first three motifs and lacked fourth motif whereas, VpvC lacked the first motif but motif 3 was present twice in VpvC (Fig. 8a). Motifs serve as signature sequences that might be useful to identify any protein. The predicted motif's e-value provides insight into the degree of functional correctness. The e-values were on the lower side for the motifs present in DGCs ranging from 9.6e^−035^ to 2.7e^−003^ (Fig. 8b). The predicted motifs are more precise with lower e-values (Sharma et al., 2022).

### 3.12 Analyses of intrinsically disordered protein regions (IDPRs)

**Fig. 9:**
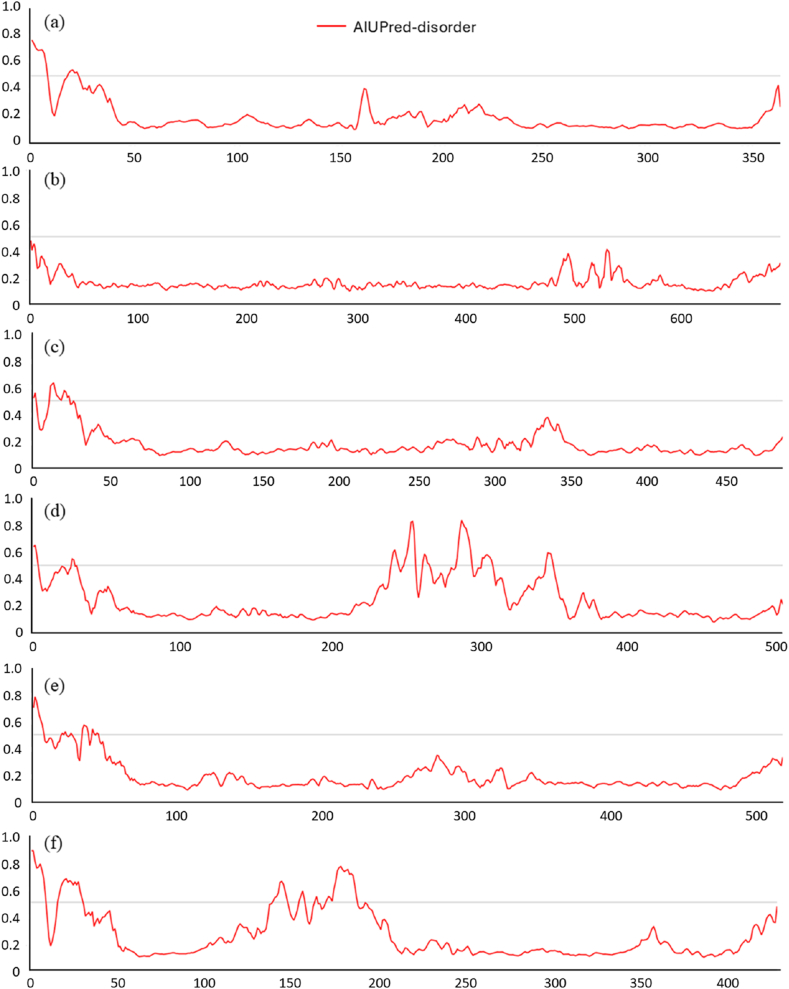
**Disordered region analyses by AIU-Pred server for DGCs:** (a) CdgA (b) CdgH (c) CdgK (d) CdgL (e) CdgM (f) VpvC. Amino acids with AIUPred scores of 0.0–0.5 are predicted to be ordered and residues 0.5–1.0 are predicted to be disordered.

Two online tools namely AIUPred and flDPnn were to determine the intrinsically disordered regions of the six studied DGCs. AIUPred used AIUPred scores to analyse IDPRs in the present investigation. Residues with score of 0.0–0.5 are predicted to be ordered and residues 0.5–1.0 are predicted to be disordered (Erdős and Dosztányi, 2024). AIUPred predicted CdgA to have a single disordered region in the stretch of 1^st^ to 9^th^ aa residues (Fig. 9a). No IDPR could be detected for CdgH (Fig. 9b). In case of CdgK the major disordered region was present at the stretch between 11^th^ and 23^rd^ aa residues (Fig. 9c). AIUPred server detected three disordered regions in CdgL comprising of aa residues in the stretches of 242^nd^-258^th^, 263^rd^-266^th^, 285^th^-310^th^ (Fig. 9d) CdgM was predicted to have 2 stretches constituting of 1^st^-8^th^ aa residues and 35^th^-46^th^ aa residues (Fig. 9e). Four small stretches of disordered residues were predicted to be present in VpvC *viz*., 1^st^-9^th^, 17^th^-30^th^, 140^th^-158^th^, 164^th^-188^th^ aa residues (Fig. 9f).

Another server flDPnn used deep feed-forward neural network to analyse physicochemical prospensities of amino acids to detect presence of disordered regions. It was revealed that CdgM had 2 % disordered regions (highest among among the studied DGCs), CdgA and CdgH were found to possess 1 % disordered residues while CdgK, CdgL and VpvC did not had disordered residues which are contrasting to results obtained by AIUPred. This might have resulted in differences in specific algorithms and cutoff points of different bioinformatic tools (Kaur et al., 2020). A former report stated that 50 % of transmembrane proteins possess at least one IDPR (Bürgi et al., 2016). However From the above findings it could be stated that that DGC structures lack major IDPRs as protein with overall disorder rate below 10–30 % is generally considered as ordered-nearly ordered protein (Avramov et al., 2022).

### 3.13 Protein-protein interactions (PPI)

**Fig. 10:**
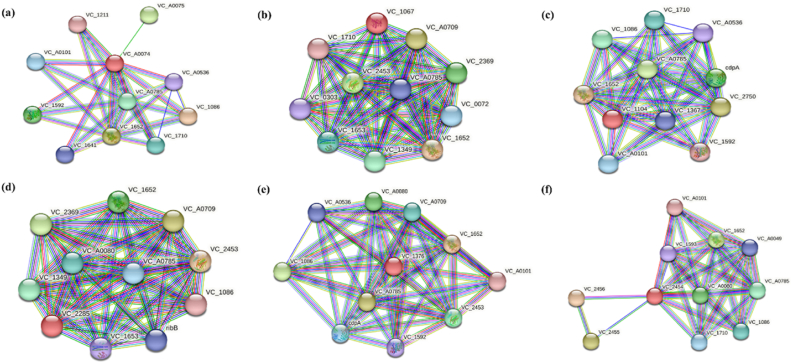
**Protein-protein interactions analysed by STRING server for DGCs:** (a) CdgA (b) CdgH (c) CdgK (d) CdgL (e) CdgM (f) VpvC.

High interaction scores for CdgA (VC_A0074) was observed with two response regulators of c-di-GMP system, *viz.*, QrgB (VC_1086), which has both GGDEF domain and EAL domain (Pursley et al., 2018) and VieA (VC_1652) with EAL domain. CdgC (VC_A0785) is another phosphodiesterase (PDE) (Conner et al., 2017) having both GGDEF domain and EAL domain was found to interact with CdgA. Other interacting proteins found to interact with CdgA in the STRING database were uncharacterized putative proteins (Fig. 10a). In case of CdgH (VC_1067) highest interaction in PPI network was observed with the EAL domain containing VieA (VC_1652). CdgH was found to interact with various histidine kinase sensor proteins like aerobic respiration control sensor protein FexB (VC_2369), response regulator VieS (VC_1653), VC_A0709, VarSA (VC_2453), VC_1349, VC_0303. VC_0303 that encodes two-component system CrbRS (Butz et al., 2021) was predicted to be co expressed with CdgH in *V. cholerae* (Fig. 10b)*.* CdgH also interacts with 2 other GGDEF domain containing proteins: VC_0072 and CdgC (VC_A0785). STRING showed interactions of CdgK (VC_1104) with VieA (VC_1652), *ibi* gene encoding (VC_2750, VC_A0709), CdpA (VC_1030), CdgC (VC_A0785), QrgB (VC_1086). In case of CdgK highest interaction was observed with VieA (VC_1652) (Fig. 10c). In case of CdgL (VC_2285) STRING network predicted high interaction scores for sensor histidine kinases *i.e.* VarSA (VC_2453), VC_A0709, FexB (VC_2369), VC_1349, VieS (VC_1653) (Fig. 10d). Its interaction with VieA was also observed. Additionally, it was found to have PPI with a RibB, which is a GTP cyclo-hydrolase (Bacher et al., 2000). CdgM (VC_1376) showed highest interaction score with VieA (VC_1652). Like the other DGCs described previously it also interacts with CdgC (VC_A0785), QrgB (VC_1086), VC_A0080, VarSA (VC_2453) and VC_A0709 (Fig. 10e). Other interacting proteins belonged to uncharacterized proteins category. For VpvC the STRING network predicted highest interaction with uncharacterized proteins VC_2456 and VC_2455 followed by VieA (VC_1652). It was also found to interact with CdgC (VC_A0785) and VC_A0080. VpvC also appeared to interact with EAL domain containing response regulator QrgB (VC_1086) (Fig. 10f). From this study it could be concluded that all the six studied DGCs have displayed high aptitudes for interactions with two PDEs, *i.e.* VieA and CdgC. So, these phosphodiesterases along with the DGCs might play important role in controlling cellular c-di-GMP levels in *V. cholerae*.

### 3.14 Molecular docking

The formation of c-di-GMP from two GTP molecules is catalysed by DGCs (Oliveira et al., 2015; Chen et al., 2021). The GG(D/E)EF domain present in the active site, so named after a conserved Gly-Gly-(Asp/Glu)-Glu-Phe sequence motif, is a diguanylate cyclase that synthesizes c-di-GMP from GTP (Whiteley and Lee, 2015). Binding of GTP to the active site of DGC results in dimerization of two DGC subunits (Chan et al., 2004). Through molecular docking the binding affinity of each DGC for GTP was studied. Additionally, the interacting residues that helps in GTP binding were also explored. Template guided molecular docking were performed using CB-DOCK2, which uses its FitDock feature to screen for similar protein structures available in the PDB and aligns the ligand in the active site to execute template guided molecular docking and the docking energies (kcal/mol) are represented as FitDock scores. FitDock has demonstrated up to 60 % improvement in docking success rates compared to conventional methods, with a significant reduction in computational time when suitable templates are available. This efficiency is achieved using hierarchical multi-feature alignment, that allows rapid exploration of possible conformations and refinement of docking poses (Yang et al., 2022). For templates with high ligand similarity (>50 %) effectiveness of template-based docking using FitDock is considered superior over the other conventional docking techniques (Yang et al., 2022). In the present study during template guided docking the ligand similarity was 100 % as the templates used in docking purpose was other DGC proteins of other bacteria bound to GTP deposited in PDB database. FitDock scores for all the studied DGCs are listed in Table 8. Contact residues of the DGCs in the DGC-GTP docked complexes performed using CB-DOCK2 match mostly with the InterPro predicted active site, indicating GTP binding occurred at the active site of the predicted 3D structures of DGCs. Similarly, in kinase drug discovery, template docking has been used to derive kinase-ligand complex data, which, when used to train graph neural networks, resulted in more precise binding affinity predictions compared to models relying solely on ligand or drug-target interaction data (Backenköhler et al., 2024).

**Table 8:** Binding energies of GTP with DGCs calculated by CD-DOCK2 and bond analysis preformed with BIOVIA-Discovery studio computational software.

*V. cholerae* DGCs	FitDock Score (kcal/mol)	Hydrogen bonds			Electrostatic bonds		Other interactions		
		No.	AA residues involved	Bond Distance (Å)	AA residues involved	Bond Distance (Å)	Type	AA residues involved	Bond Distance (Å)
CdgA	−3.8	3	Asn252	2.56, 2.46, 2.43	Asp288	4.23	Hydrophobic	Gly287	3.58
			Asp261	1.93	Asp261	3.04			
			Arg284	2.47					
CdgH	−4.8	3	Asn573	2.12, 2.36, 2.57	Asp582	2.14			
			His578	2.22					
			Asp582	2.56					
CdgK	−4.4	3	Asn376	3.49	Asp385	3.66			
			His381	3.71	Glu411	4.21			
			Asp385	3.72					
CdgL	−5.5	4	Asn383	2.30	Asp392	3.28			
			His388	2.52					
			Asp392	3.35	Glu419	4.43			
			Gly418	2.97					
CdgM	−4.8	3	Asn402	2.27, 2.52, 2.67	Asp411	2.23	C-H bond	Lys399	3.49
			His407	2.06				Gly436	3.70
			Asp411	2.55					
VpvC	−5.6	5	Lys287	3.02	Asp282	3.56	C-H bond	Lys287	3.53
			Asn290	2.44,2.29	Asp299	2.43			
			His295	2.68	Asp328	3.74, 4.93			
			Asp299	2.38	Glu329	4.35			
			Arg324	3.02					

**Fig. 11:**
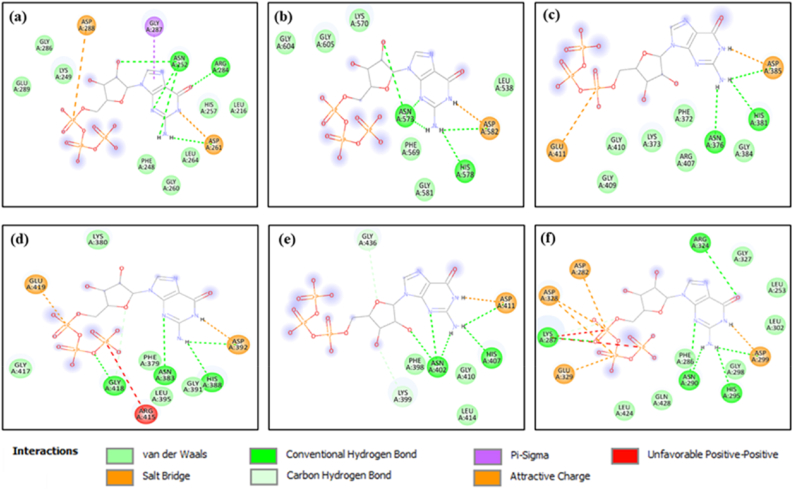
**Molecular docking studies on the interaction of DGCs with GTP:** Protein-ligand interaction obtained by CB-DOCK 2 were visualised using Discovery studio software for CdgA, CdgH, CdgK, CdgL, CdgM, VpvC respectively (a, b, c, d, e, f).

The binding affinity of CdgA to GTP was found to be −3.8 kcal/mol and the interacting residues were Leu216, Phe248, Lys249, Asn252, His257, Gly260, Asp261, Leu264, Arg284, Gly286, Gly287, Asp288 and Glu289. Interestingly it was found among all the DGC-GTP complexes, hydrophobic interaction was found only in CdgA-GTP complex (Table 8). It was observed that three H-bonds and two electrostatic interactions were responsible for the complex stability (Fig. 11a). In case of CdgH calculated binding energy was −4.8 kcal/mol and contact residues of the protein-ligand interaction were Leu538, Phe569, Lys570, Asn573, Gly577, His578, Glu579, Gly581, Asp582, Arg602, Gly604, Gly605 and Asp606. Similar to afore mentioned CdgA-GTP complex here also three H-bonds were formed between CdgH and GTP, but contrastingly only one electrostatic interaction could be found here (Fig. 11b). Molecular docking of CdgK with GTP revealed the binding affinity of the former to the later to be −4.4 kcal/mol and the contact residues were Phe372, Lys373, Asn376, Gly380, His381, Asp382, Gly384, Asp385, Phe408, Gly409, Gly410 and Glu411. Three H-bonds and two electrostatic interactions added stability to the complex (Fig. 11c). The GTP binding template used by CB-DOCK 2 tool for CdgA, CdgH, CdgK was PleD (PDB:2WB4), a studied DGC from *Caulobacter vibrioides* (Wassmann et al., 2007). The binding affinity of CdgL with GTP calculated by CB-DOCK2 was −5.5 kcal/mol and the contact residues were found to be Asp342, Phe379, Lys380, Asn383, His388, Leu389, Gly391, Asp392, Leu395, Arg415, Phe416, Gly417, Gly418 and Glu419. Interestingly it was observed four H-bonds were found in the complex. In addition, two electrostatic interactions along with an unusual positive-positive interaction (mediated by Arg415) were also featured here (Fig. 11d). The template used for this docking interaction was a DGC of *Escherichia coli* K12 DgcZ (PDB ID:3TVK) (Zähringer et al., 2013). Binding energy for CdgM and GTP was predicted to be −4.8 kcal/mol and the contact residues were Phe398, Lys399, Asn402, Gly406, His407, Leu408, Ala409, Gly410, Asp411, Leu414, Gly435, Gly436 and Asp437. Docking studies deciphered two C-H bonds mediated by Lys399 and Gly436 along with three H-bonds and a single electrostatic interaction was responsible for CdgM-GTP binding (Fig. 11e). PleD (Wassmann et al., 2007) was used for the template guided docking of CdgM. VpvC displayed a binding energy of – 5.6 kcal/mol for the template guided docking with contact residues being Leu253, Asp282, Phe286, Lys287, Asn290, Gly294, His295, Ala296, Gly298, Asp299, Leu302, Arg324, Gly326 Gly327, Asp328, Glu329, Lys399, Leu424 and Gln428. The highest number of interactions was observed among VpvC and GTP *i.e.* five H-bonds, four electrostatic interactions, one C-H bond (Fig. 11f). As a result, highest docking score was observed for VpvC among all studied DGCs for GTP binding. A recent study unveiled that VpvC plays substantial role in contributing to high biofilm forming ability in *V. cholerae* (Manna et al., 2024). *E. coli* DgcZ (PDB ID:4H54) was used as template reference to study VpvC-GTP interaction (Zähringer et al., 2013). It was previously deciphered that Asp(D) in the GGDEF domain in the DGC JcaA is responsible for deprotonation of 3′-hydroxyl group of ribose sugar present in GTP resulting in nucleophilic attack on alpha phosphate of target GTP molecule in *Candidatus* sp (Guo et al., 2017). Thus, it might be predicted that the amino acid residue responsible for deprotonation of 3′-OH of GTP ribose sugar are Asp288, Asp606, Asp437, Asp328 in case of CdgA, CdgH, CdgM, VpvC, respectively. However, the Asp in the active site is replaced by Glu in CdgK and CdgL, resulting in GGEEF active site. So, in these DGCs the Glu(E) residing at the third position of GGEEF moiety might be responsible for nucleophilic attack on GTP. This docking study unveiled that among all 6 studied DGCs of *V. cholerae* VpvC had highest affinity for GTP, and it was closely followed by CdgL. Another significant finding observed in the study is Asn residue (252nd in CdgA, 573rd in CdgH, 376th in CdgK, 383rd in CdgL, 402nd in CdgM, 290th in VpvC) residing near the signature GGDEF domains in all studied DGCs plays key role in incorporation of the GTP in the DGC active site. Prior studies on GTP binding in active sites of target receptors reported Asn is responsible for H-bonding with the guanine ring of GTP. In *Campylobacter jejuni* substitution of Asn128 in G4 motif of GTP-binding domain caused reduced GTP binding ability (Grewal et al., 1993). Hence it could be suggested Asn in DGC active sites may play similar role and strategies might be developed to inhibit the activity of Asn residues to design novel anti-biofilm drugs for future cholera management strategies.

### 3.15 MM/GBSA calculations

MM/GBSA is a computational method used to estimate changes in free energy of binding in biomolecular systems. Present observations revealed VpvC and CdgL exhibited lowest binding energies in MM/GBSA (−37.025 and −34.151 kcal/mol) corresponding to its higher affinity towards GTP (Table 9). Results obtained hereby complemented the molecular docking studies in terms of binding affinity of DGCs with GTP. Higher number of H-bonds might be responsible for the corresponding low MM/GBSA values (Ghosh et al., 2021).

**Table 9:** MM/GBSA and Eigen values of the DGC-GTP complexes.

DGCs	MM/GBSA (kcal/mol)	10^6^ x Eigen value
CdgA	−27.561	1.075372
CdgH	−28.385	2.552371
CdgK	−27.752	1.147114
CdgL	−32.025	0.441004
CdgM	−27.386	6.316077
VpvC	−34.151	0.469564

### 3.16 Molecular dynamics (MD) simulation studies

**Fig. 12:**
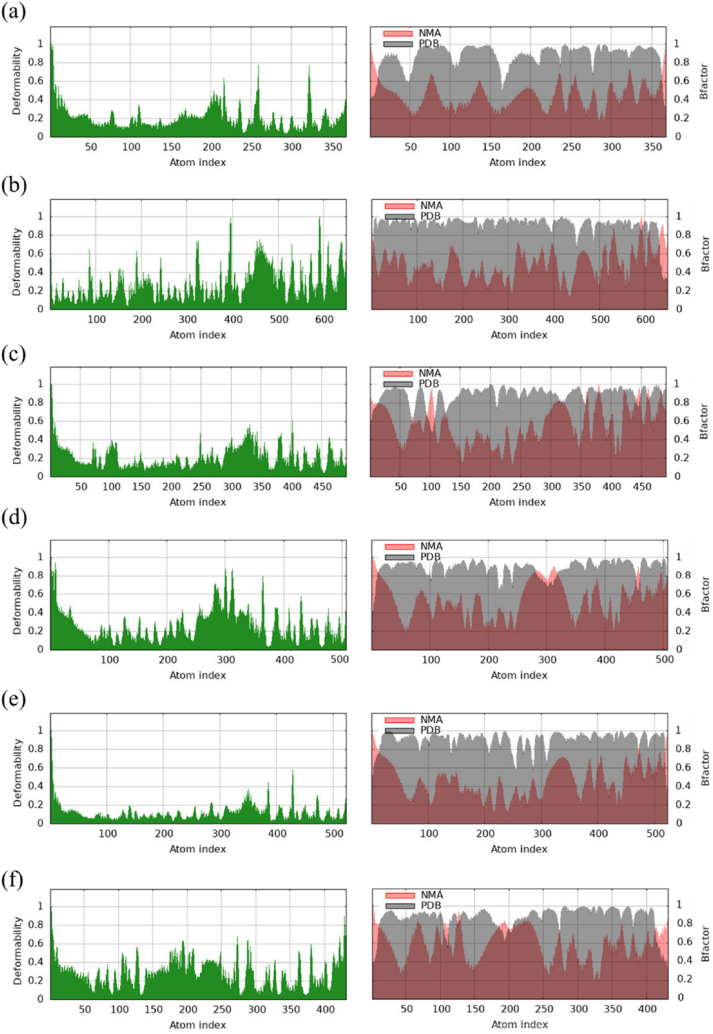
**Deformability and B-factors of the studied DGCs analysed by MD simulations using iMODS server:** (a) CdgA, (b) CdgH, (c) CdgK, (d) CdgL, (e) CdgM and (f) VpvC.

iMODS run used normal mode analysis (NMA) to investigate the dynamics of the docked complexes and to demonstrate their large-amplitude conformational fluctuations (Sumera et al., 2022). Deformability and B-factor helped to analyse mobility profiles of docked complex. Peaks close to 1 in deformabiliity graph (Fig. 12) represents regions in the docked complex with high flexibilities (Zia et al., 2024). Fig. 12b clearly reveals that the docked CdgH-GTP complex possessed hinges/peaks with the amplitudes close to 1 which indicate that among the studied DGCs it had high structural flexibility whereas CdgM-GTP hinges (Fig. 12e.) of lower amplitudes (up to 0.6) indicate high structural rigidity of the docked complex. B-factors graph described the comparison of PDB and NMA field of the receptor and receptor-ligand complexes. In all DGCs patterns of peaks obtained from NMA simulations were quite similar with PDB peaks meant the interaction patterns might be similar with experimental results (Pokharkar et al., 2022). Eigenvalue obtained from the simulations represents the stability of docked complexes. It corresponds to the required energy to deform the structure and low eigenvalues are associated with high structural stability (Pokharkar et al., 2022; Zia et al., 2024). Eigenvalues of the DGC-GTP docked complexes are listed in Table 9.

It could be interpreted from the aforementioned information that the studied complexes were stable as the computed eigenvalues of the complexes were lower than previously reported ligand-protein interactions with good stability (Pokharkar et al., 2022; Sumera et al., 2022). Eigenvalues are inversely related with variance. It was observed in Fig. S4 (Supplementary section) that CdgL and VpvC with low eigenvalues had high isolated variance (≈50 %) indicating their greater stability among the studied complexes.

## 4 Conclusions

In cholera management, oral vaccines are costly, provide temporary solution, and sustainable long-term solutions remain elusive which eventually require alternative strategies. Given that, biofilm is the primary mode of survival for *V. cholerae* in both human gut and aquatic environments, targeting the pathogen's biofilm may serve as one of the viable alternative strategies.

DGCs are GGDEF domain containing proteins that catalyse the formation of c-di-GMP from GTP molecules in bacterial systems and essential for biofilm formation. In the present study different bioinformatic tools were exploited to have preliminary insights on genomic features and structural variations in six major biofilm regulatory DGCs of *V. cholerae*. Genomic studies revealed that natural selection plays superior role over mutational pressure in shaping the codon usage patterns, thereby suggesting ideal candidature of these DGCs as drug targets. Codon adaptation index values over 0.6 in all DGCs indicated their high adaptation and expression probability in diverse environmental milieu. All six DGCs were predicted to be transmembrane proteins. Physicochemical parameters revealed that among the studied DGCs, CdgK, CdgL, and CdgM might exhibit low stability beyond cellular environments. Secondary structural studies suggested α-helix to be the predominant secondary structural feature and leucine is the most abundant amino acid in studied DGCs. Tertiary structure prediction and molecular docking analyses of the DGCs with GTP helped to identify the crucial active site amino acid residues involved in ligand binding. The present study is considered to have potential in designing advance-level experiments to decipher structural properties of these DGCs. Moreover, it could also be beneficial in identifying and/or designing novel DGC inhibitors that target and down-regulate the c-di-GMP regulatory cascade, leading to the development of innovative anti-biofilm agents against the cholera pathogen. However, further detailed analytical methodologies are necessary to substantiate the theoretical information gathered herein. Cryo-electron microscopy and other advanced structural biology techniques may be useful to validate current findings and comprehend the physicochemical characteristics of these proteins.

## CRediT authorship statement

Tuhin Manna: Conceptualization, investigation, methodology, data analysis, and writing original manuscript. Subhamoy Dey: Methodology and formal analysis. Monalisha Karmakar: Formal analysis and editing manuscript. Amiya Kumar Panda: Supervision, reviewed and edited manuscript and Chandradipa Ghosh: Conceptualization, Supervision, reviewed and edited manuscript.

## Data availability statement

All relevant data are within the paper and its Supporting Information files**.**

## Funding

Tuhin Manna, Subhamoy Dey, Monalisha Karmakar acknowledge University Grants Commission (10.13039/501100001501UGC), New Delhi, India for providing fellowships to carry out research work.

## Declaration of competing interest

The authors declare that they have no known competing financial interests or personal relationships that could have appeared to influence the work reported in this paper.

## Data Availability

Data will be made available on request.
