# Supplementary material for: Investigations on genomic, topological and structural properties of diguanylate cyclases involved in Vibrio cholerae biofilm signalling using in silico techniques: Promising drug targets in combating cholera
Source: Curr Res Struct Biol. 2025 Apr 9;9:100166. doi: 10.1016/j.crstbi.2025.100166 (PMC12051071; doi:10.1016/j.crstbi.2025.100166)
Supplement: Multimedia component 1 [file mmc1.docx]

**Investigations on genomic, topological and structural properties of diguanylate cyclases involved in *Vibrio cholerae* biofilm signalling using *in silico* techniques: Promising drug targets in combating cholera**

Supplementary Section

Tuhin Manna^1^, Subhamoy Dey^1,3^, Monalisha Karmakar^1^, Amiya Kumar Panda^2^, Chandradipa Ghosh^1*^

^1^Deparment of Human Physiology, Vidyasagar University, Midnapore, West Bengal, India

^2^Department of Chemistry, Vidyasagar University, Midnapore, West Bengal, India

^3^Centre for Life Sciences, Vidyasagar University, Midnapore, West Bengal, India

Author for correspondence:

Professor Chandradipa Ghosh

Department of Human Physiology

Vidyasagar University

Midnapore, West Bengal-721102

India

Email: ch_ghosh@mail.vidyasagar.ac.in

Table S1. RSCU analysis of each amino acid in studied *V. cholerae* DGC genes

| Amino acids | Codons | CdgA | CdgH | CdgK | CdgL | CdgM | VpvC |
| --- | --- | --- | --- | --- | --- | --- | --- |
| Phe | UUU | **1.15** | **1.5** | **1.81** | **1.09** | **1.38** | 0.93 |
|  | UUC | 0.85 | 0.5 | 0.19 | 0.91 | 0.62 | **1.07** |
| Leu | UUA | 0 | 0.96 | 1.5 | 0.11 | 1.29 | 0 |
|  | UUG | **2.06** | **1.41** | 1.15 | **1.92** | 1.11 | 1.29 |
|  | CUU | 0.34 | 1.19 | 0.58 | 0.68 | 0.65 | 0.71 |
|  | CUC | 1.89 | 0.89 | 1.04 | 1.81 | 0.55 | 1.88 |
|  | CUA | 0 | 0.81 | 0.23 | 0 | 0.37 | 0.12 |
|  | CUG | 1.71 | 0.74 | **1.5** | 1.47 | **2.03** | **2** |
| Ile | AUU | **1.11** | **1.43** | **1.54** | 0.74 | **1.69** | 0.69 |
|  | AUC | **1.11** | 1.14 | 1.08 | 1.42 | 1.12 | **1.69** |
|  | AUA | 0.78 | 0.43 | 0.38 | 0.85 | 0.19 | 0.62 |
| Val | GUU | **1.8** | 1.02 | 1.49 | 0.89 | 1.07 | 1.11 |
|  | GUC | 0.8 | 0.63 | 0.34 | **1.33** | 0.8 | 0.67 |
|  | GUA | 0.8 | 0.55 | 0.57 | 0.89 | 0.27 | **1.56** |
|  | GUG | 0.6 | **1.8** | **1.6** | 0.89 | **1.87** | 0.67 |
| Ser | UCU | 0.38 | 0.82 | 0.32 | 0.24 | 0.6 | 0.67 |
|  | UCC | 1.5 | 0.27 | 0.81 | 1.2 | 0.45 | 1 |
|  | UCA | 0.38 | 0.95 | 0.65 | 0 | 1.05 | 0 |
|  | UCG | 0.38 | 0.95 | 0.65 | 1.2 | **1.35** | 1 |
|  | AGU | **3** | **1.64** | 1.62 | 0.96 | 1.2 | 1.33 |
|  | AGC | 0.38 | 1.36 | **1.95** | **2.4** | **1.35** | **2** |
| Pro | CCU | 0 | **1.2** | **1.11** | 0 | 1.18 | 0 |
|  | CCC | **2** | 0.6 | 0.89 | 1.67 | 0.47 | **1.45** |
|  | CCA | 1.33 | **1.2** | **1.11** | **2** | 0.94 | **1.45** |
|  | CCG | 0.67 | 1 | 0.89 | 0.33 | **1.41** | 1.09 |
| Thr | ACU | **2** | 1.07 | 0.63 | 1.07 | **1.48** | 1.19 |
|  | ACC | 0.67 | **1.6** | **1.26** | 0.8 | 1.33 | **1.33** |
|  | ACA | 0.67 | 0.4 | 1.05 | 0.8 | 0.44 | 0.3 |
|  | ACG | 0.67 | 0.93 | 1.05 | **1.33** | 0.74 | 1.19 |
| Ala | GCU | **1.73** | **1.49** | 0.95 | **1.64** | 0.5 | 0.76 |
|  | GCC | 0.67 | 0.78 | **1.37** | 1.27 | 0.83 | **1.14** |
|  | GCA | 0 | 0.47 | 0.63 | 0.18 | 1.17 | 0.19 |
|  | GCG | 1.6 | 1.25 | 1.05 | 0.91 | **1.5** | 1.9 |
| Tyr | UAU | **1** | **1.14** | **1.46** | 0 | **1.1** | 0.8 |
|  | UAC | **1** | 0.86 | 0.54 | **2** | 0.9 | **1.2** |
| His | CAU | **1.5** | 0.88 | **1.57** | 0.69 | **1.5** | 0.73 |
|  | CAC | 0.5 | **1.12** | 0.43 | **1.31** | 0.5 | **1.27** |
| Gln | CAA | 0.92 | 0.97 | 0.8 | **1.4** | **1.22** | 0.91 |
|  | CAG | **1.08** | **1.03** | **1.2** | 0.6 | 0.78 | **1.09** |
| Asn | AAU | **1.67** | 0.93 | **1.71** | **1.12** | **1.06** | **1.59** |
|  | AAC | 0.33 | **1.07** | 0.29 | 0.88 | 0.94 | 0.41 |
| Lys | AAA | **1.76** | 1.24 | **1.5** | **1.6** | **1.83** | 1.22 |
|  | AAG | 0.24 | 0.76 | 0.5 | 0.4 | 0.17 | 0.78 |
| Asp | GAU | 1.57 | **1.29** | **1.5** | 1.2 | 1.57 | **1.56** |
|  | GAC | 0.43 | 0.71 | 0.5 | 0.8 | 0.43 | 0.44 |
| Glu | GAA | **1.05** | **1.24** | **1.11** | **1.26** | 0.92 | 0.57 |
|  | GAG | 0.95 | 0.76 | 0.89 | 0.74 | **1.08** | **1.43** |
| Cys | UGU | **1.43** | **1.5** | 0.4 | 0.53 | **1.5** | 0.5 |
|  | UGC | 0.57 | 0.5 | **1.6** | **1.47** | 0.5 | **1.5** |
| Arg | CGU | 0.96 | **2.63** | 1.75 | 0.95 | **2.23** | 0.89 |
|  | CGC | **1.68** | 1.9 | **3.25** | **1.89** | 1.89 | **2.89** |
|  | CGA | 1.44 | 0.59 | 0.25 | 0.95 | 1.03 | 0.44 |
|  | CGG | 0.48 | 0.59 | 0 | 0.95 | 0.51 | 0.67 |
|  | AGA | 0.96 | 0.29 | 0.75 | 0.95 | 0 | 0.89 |
|  | AGG | 0.48 | 0 | 0 | 0.32 | 0.34 | 0.22 |
| Gly | GGU | 0.76 | **1.42** | **2** | 1.12 | **1.39** | 0.8 |
|  | GGC | **2.48** | 1.03 | 0.73 | **1.6** | 1.04 | **1.87** |
|  | GGA | 0.19 | 0.65 | 0.18 | 0.64 | 0.52 | 0.8 |
|  | GGG | 0.57 | 0.9 | 1.09 | 0.64 | 1.04 | 0.53 |


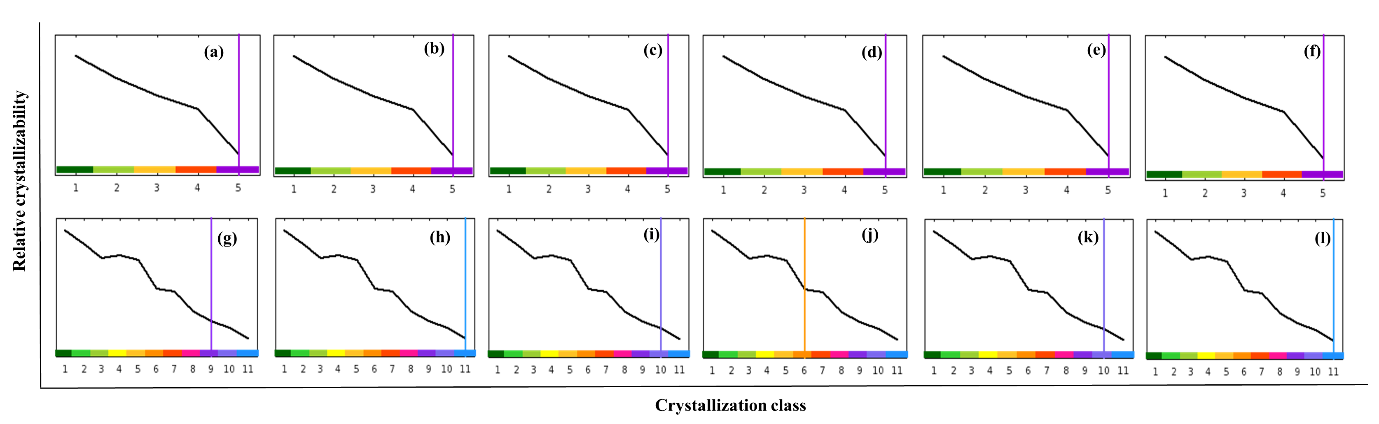


**Fig S1. Crystallization scores of DGCs:** EP crystallization scores of CdgA, CdgH, CdgK, CdgL, CdgM, VpvC were presented respectively (a-f). All the DGCs had scored 5 in EP crystallization technique. RF crystallization scores of CdgA, CdgH, CdgK, CdgL, CdgM, VpvC were 9, 11, 10, 6, 10,11 respectively (g-l).


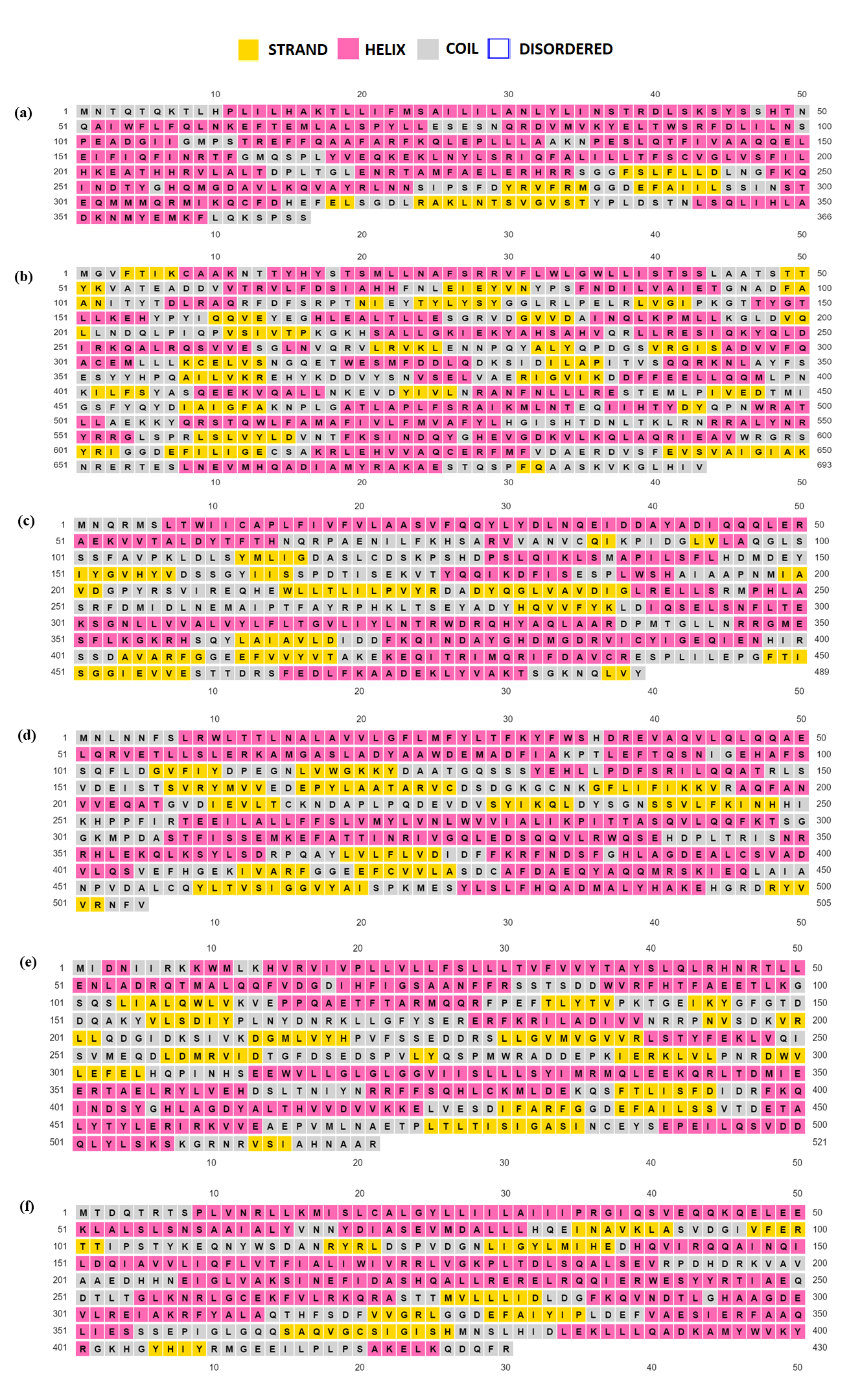


**Fig S2.** **Sequence of** **individual amino acids participating in secondary structure formation of DGCs, as analysed by PSIPRED:** (a) CdgA (b) CdgH (c) CdgK (d) CdgL (e) CdgM (f) VpvC.


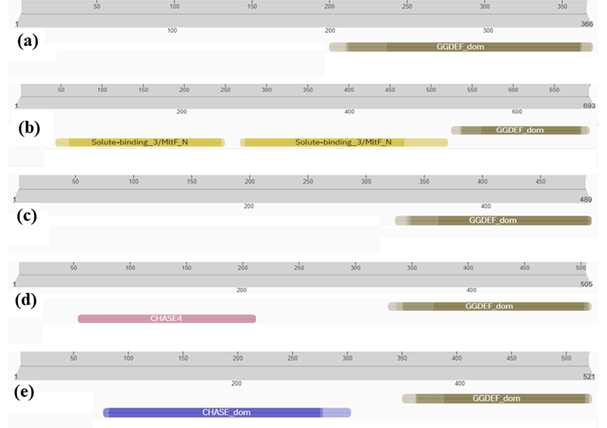


**Fig S3.** **Predominant domains present in DGCs:** (a) CdgA possess GTP binding GGDEF domain; (b) CdgH with two solute binding domains and third GGDEF domain; (c) CdgK having single GGDEF domain; (d) CdgL with CHASE 4 and GGDEF domain (e) CdgK having a CHASE domain and another GGGDEF domain; (f) VpvC having a unique HAMP domain and the GGDEF domain.


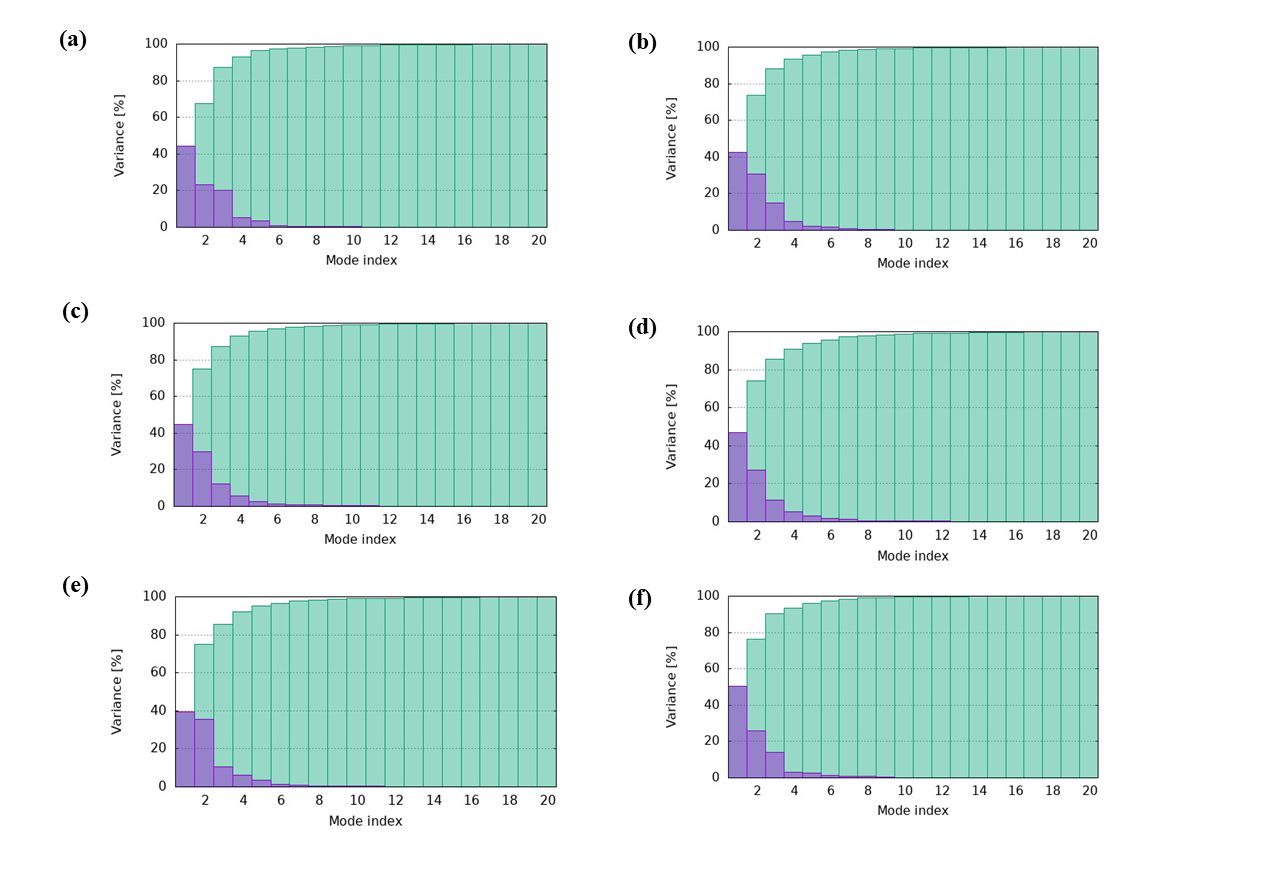


**Fig S4. Variance plots of DGCs:** Displayed bars of purple colour represent isolated variance, while cumulative variance is indicated by green coloured bars for (a) CdgA (b) CdgH (c) CdgK (d) CdgL (e) CdgM (f) VpvC.
